# Isolation and In Silico Prediction of Potential Drug-like Compounds with a New Dimeric Prenylated Quinolone Alkaloid from *Zanthoxylum rhetsa* (Roxb.) Root Extracts Targeted against SARS-CoV-2 (Mpro)

**DOI:** 10.3390/molecules27238191

**Published:** 2022-11-24

**Authors:** Fatema Tuz Zohora, A. T. M. Zafrul Azam, Sinthyia Ahmed, Khondaker Miraz Rahman, Mohammad A. Halim, Md. Rafi Anwar, Md. Hossain Sohrab, Fatema Tabassum, Choudhury Mahmood Hasan, Monira Ahsan

**Affiliations:** 1 Department of Pharmaceutical Chemistry, University of Dhaka, Dhaka 1000, Bangladesh; 2 Department of Pharmacy, University of Asia Pacific, 74/A, Green Road, Dhaka 1205, Bangladesh; 3 Division of Computer Aided Drug Design, The Red-Green Research Centre, BICCB, Tejgaon, Dhaka 1219, Bangladesh; 4 School of Cancer and Pharmaceutical Science, King’s College London, 150 Stamford Street, London SE1 9NH, UK; 5 Department of Chemistry and Biochemistry, Kennesaw State University, Kennesaw, GA 30144, USA; 6 College of Pharmacy, University of Louisiana at Monroe, Monroe, LA 71201, USA; 7 Bangladesh Council of Scientific and Industrial Research (BCSIR), Dr. Qudrat-E-Khuda Road, Dhaka 1205, Bangladesh; 8 Department of Pharmacy, Stamford University Bangladesh, 51 Siddheswari Rd, Dhaka 1217, Bangladesh

**Keywords:** *Zanthoxylum rhetsa*, 2,11-didemethoxy-vepridimerine A, SARS-CoV-2 (Mpro), 2-quinolone, benzophenanthridine alkaloids

## Abstract

A new dimeric prenylated quinolone alkaloid, named 2,11-didemethoxy-vepridimerine A, was isolated from the root bark of *Zanthoxylum rhetsa*, together with twelve known compounds. The structure of the new compound was elucidated on the basis of spectroscopic investigations (NMR and Mass). The interaction of the isolated compounds with the main protease of SARS-CoV-2 (Mpro) was evaluated using molecular docking followed by MD simulations. The result suggests that 2,11-didemethoxy-vepridimerine A, the new compound, has the highest negative binding affinity against the Mpro with a free energy of binding of −8.5 Kcal/mol, indicating interaction with the Mpro. This interaction was further validated by 100 ns MD simulation. This implies that the isolated new compound, which can be employed as a lead compound for an Mpro-targeting drug discovery program, may be able to block the action of Mpro.

## 1. Introduction

Viral diseases initiated by the coronavirus (CoV) have become one of the major public health problems worldwide in the last two decades. COVID-19 is caused by SARS-CoV-2, which is a highly contagious novel coronavirus. The recent emergence of the deadly COVID-19 due to SARS-CoV-2 has created unprecedented pandemic situations around the globe, increasing the need for antiviral molecules to treat them [1,2].

There is an urgent clinical need to find new antiviral agents against SARS-CoV-2 as, except for the recently approved Paxlovid from Pfizer and some repurposed drugs like remdesivir and favipiravir, there is no approved direct acting antiviral drug that works against SARS-CoV-2 targets.

Natural products and herbal medicines provide a unique dimension in the drug development methods for antiviral medication. From a drug discovery point of view, active ingredients extracted from natural products usually possess exceptional novelty in structures with intrinsic pharmacological activities. Many studies on natural products with an antiviral effect shed light on some bioactive ingredients’ potential against various viral diseases [1,3,4]. The effectiveness of computationally-assisted drug design as one of the versatile tools for facilitating drug discovery and development has been recognized for decades, especially in the case of natural products [4,5,6]. A number of plant secondary metabolites such as glycyrrhizin, baicalin, and quercetin have been studied in silico and were found to have antiviral and anti-SARS-CoV activity [7].

The family Rutaceae is known for generating a large variety of secondary metabolites like phenanthridine, acridone and furo- and pyranoquinoline alkaloids, complicated furo- and pyranocoumarins, flavonoids, and numerous sorts of terpenoids, together with the limonoids [8], that have shown a range of pharmacological activities. In contrast, the SARS-CoV-2 genomes call for 16 non-structural proteins, one of which is the major protease (Mpro), which is in charge of replicating proteins and stopping viral replication [9]. Alkaloids are employed against viruses as antiviral agents. The isoquinoline alkaloids tetrandrine, fangchinoline, and cepharanthine have been shown in studies to have the ability to treat HCoV-OC43 infection [9]. The importance of SARS-CoV-2 and HCoV-OC43 cocirculation has been drawn into attention, especially during the COVID-19 pandemic. Since there might be cross-reactivity between SARS-CoV-2 and endemic coronaviruses such as HCoV-OC43, diagnostic challenges are proposed and should be well addressed in laboratory approaches. Interestingly, it has been demonstrated that elevated levels of HCoV-OC43 S-IgG can be found in SARS-CoV-2 patients, and the titer is associated with the severity of the disease [9,10]. The isoquinoline alkaloid palmatine inhibits the growth of the West Nile and Zika viruses. Sanguinarine is used for antibacterial activities and exhibits antiviral activity against the human immunodeficiency virus protease and the herpes simplex virus. Chelidonine exhibits antiviral impact against the herpes simplex virus, human immunodeficiency virus, and the influenza virus. Emetine is a potential SARS-CoV-2 antiviral agent [11,12]. On the other side, *Z*. *rhetsa* is recognized as not only a spice, but also as a medicinal plant which has been popularly used in many tropical countries such as Vietnam, Thailand, and Bangladesh. Phytocompounds from parts of this plant were reported with extra health benefits, including antinociceptive and antidiarrheal, antioxidant, antimalarial, antimicrobial, and antibacterial activities. In addition, essential oils from *Z. rhetsa* fruits have shown a preventative ability on several breast and lung cancer cell lines, as well as on leukemia [13]. Referring to such recent studies, this study primarily demonstrates the isolation of a new dimeric prenylated quinolone alkaloid named 2,11-didemethoxy-vepridimerine A, together with twelve known compounds that are alkaloids, lignan and steroids from the root bark of *Zanthoxylum rhetsa* (Roxb.) DC. belonging to the Rutaceae family. It evaluates their ability to inhibit the SARS-CoV-2 Mpro by in silico technique, and molecular docking was performed using the AutoDock Vina software. Molecular dynamic simulations were also performed for the new compound **1**, along with the apo (the unbound protein as apo structure) form [14]. 

## 2. Results

**Figure 1:**
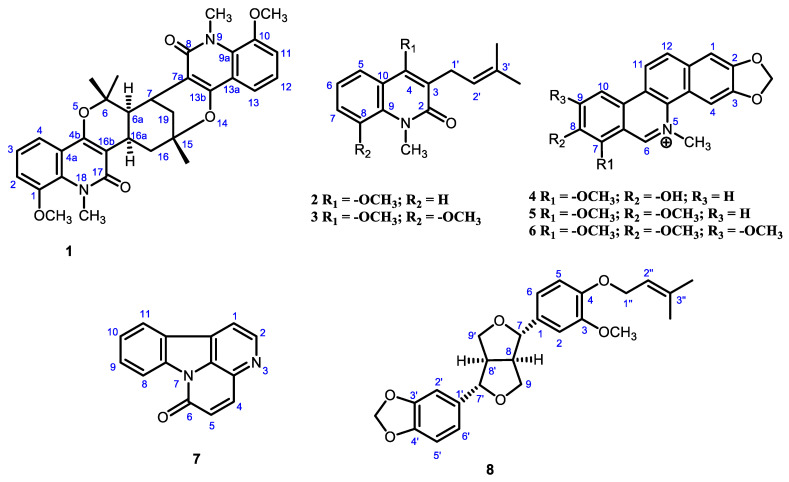
Structures of the compounds isolated from *Zanthoxylum rhetsa*.

A methanol extract of the root bark of *Zanthoxylum rhetsa* afforded thirteen chemical compounds. Among them, 2,11-didemethoxy-vepridimerine A (**1**) is a new dimeric prenylated quinolone alkaloid, N-methylatanine (**2**), and 3-dimethylallyl-4,8-dimethoxy-1-methyl-2-quinolone (**3**) 2-quinolone alkaloids isolated for the first time from the genus Zanthoxylum. 8-O-demethylchelerythrine (**4**), chelerythrine (**5**) earlier isolated from *Z. rhetsa* and 7-methoxynitidine (**6**) also appeared as a new natural compound, however it was synthesized previously, canthin-6-one (**7**), (+)-piperitol-γ-γ-dimethylallylether (**8**). stigmasterol (**9**), β-sitosterol (**10**), methyl oleate (**11**), methyl stearate (**12**) & stearic acid (**13**). A very common structure (Figure 1) was also isolated and identified by comparison of their spectroscopic data with those reported in the literature.

### 2.1. Characterization of Compounds

**Figure 2:**
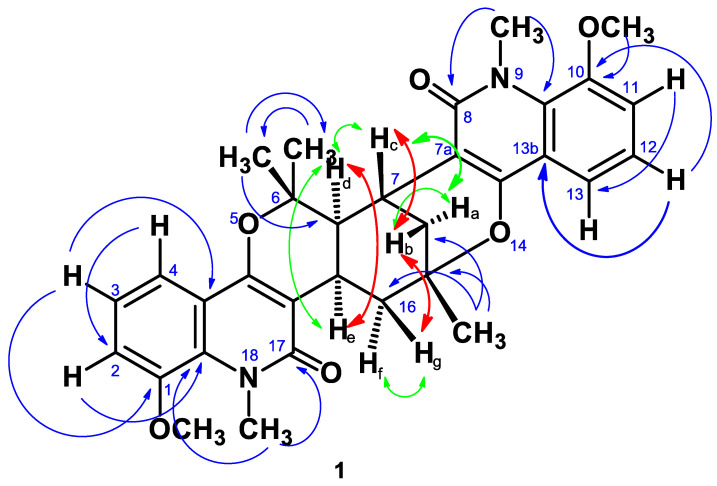
Key HMBC (blue), COSY (green) and NOESY (red) correlations for Compound **1**.

Compound **1**, isolated as a yellowish mass, showed a blue fluorescent and quenching spot when examined under 366 nm and 254 nm UV light on a TLC plate. It produced a very light-yellow color when sprayed with vanillin in sulphuric acid reagent followed by heating for 5 min. It gave an orange red color after spraying with Dragendorff’s reagent. The HRESIMS showed a peak at m/z 543.2480 (MH^+^) measured in the positive ion mode and solved for the molecular formula C_32_H_34_N_2_O_6_. The ^1^H NMR spectrum (400 MHz, CDCl_3_; Table 1, Figure S1) revealed two pairs of three adjacent aromatic protons at δ 7.05 dd (H-2), 7.17 dd (H-3), 7.63 dd (H-4) and δ 7.04 dd (H-11), 7.14 dd (H-12), 7.73 dd (H-13), two methoxy groups at δ 3.89 and 3.88 ppm, and two N-methyls at δ 3.93 and 3.88 ppm. In addition, the spectrum displayed three tertiary methyls at δ 1.55 (Me-6), 1.87 (Me-6), and 1.55 (Me-15) ppm in addition to seven aliphatic protons at δ 1.57 to 3.70 ppm (Ha-Hg) (Table 1). The ^13^C NMR spectrum (Table 1, Figure S2) showed thirty-two carbons altogether, including two carbonyl carbons at δ 163.9 and 163.0 ppm. The ^1^H and ^13^C NMR spectrum, jointly with the HSQC (Figure S4) spectrum, revealed carbons assigned to six aromatic methines, three aliphatic methines, two methylenes, two methoxyls, two N-methyls, three tertiary methyls, four saturated quaternary carbons (two being oxygenated) and eight aromatic/olefinic quaternary carbons. All these data were similar to those of vepridimerine A [15,16], except for the absence of two methoxyl groups at C-2 and C-11. That is, compound **1** consists of two 8-methoxy-N-methyl quinolone units joined by a C_10_ moiety. The small coupling constant of 6.3 Hz between H_d_ and H_e_ suggested a cis relationship as in vepridimerine A (Table 1 and Table 2). The COSY (Figure S3), HSQC, and HMBC (Figure S5) spectra allowed assignment of all the carbons and protons in the molecule. The relative stereochemistry was confirmed by a NOESY (Figures S6 and S7) experiment which showed cross-peaks between H_d_ & H_e_, and also between H_b_ & H_c_ and H_b_ & H_g_ (Figure 2). On the basis of the above data, compound **1** was characterized as 2,11-didemethoxy-vepridimerine A. This is the first report of the isolation of this alkaloid from a natural source.

Compound (**2**), ^1^H NMR (400 MHz, CDCl_3_): 7.76 dd (*J* = 8.0, 1.1 Hz, H-5), 7.20 ddd (*J* = 8.6, 8.0, 1.4 Hz, H-6), 7.48 ddd (*J* = 8.6, 8.4, 1.1 Hz, H-7), 7.30 d (*J =* 8.4 Hz, H-8), 3.34 d (2H, *J =* 7.0 Hz, H-1′), 5.20 t (*J =* 7.0 Hz, H-2′), 1.62, 3H s (Me-3′cis), 1.75, 3H s (Me-3′trans), 3.66, 3H s (N-Me), 3.85 3H s (OMe-4). ^13^C (100 MHz, CDCl_3_): 164.0 (C-2), 122.6 (C-3), 160.2 (C-4), 123.4 (C-5), 121.9 (C-6), 130.1 (C-7), 114.1 (C-8), 139.0 (C-9), 122.4 (C-10), 24.3 (C-1′), 121.5 (C-2′), 132.5 (C-3′), 25.7 (Me-3′cis), 19.0 (Me-3′trans) 29.8 (N-Me), 61.8 (OMe-4) [17].

Compound (**3**), ^1^H NMR (400 MHz, CDCl_3_): 7.76 dd (*J =* 8.0, 1.1 Hz, H-5), 7.20 ddd (*J =* 8.6, 8.0, 1.4 Hz, H-6), 7.48 ddd (*J =* 8.6, 8.4, 1.1 Hz, H-7), 7.30 d (*J =* 8.4 Hz, H-8), 3.34 d (2H, *J =* 7.0 Hz, H-1′), 5.20 t (*J =* 7.0 Hz, H-2′), 1.62, 3H s (Me-3′cis), 1.75, 3H s (Me-3′trans), 3.66, 3H s (N-Me), 3.85, 3H s (OMe-4). ^13^C (100 MHz, CDCl_3_): 164.0 (H-2), 122.6 (H-3), 160.2 (H-4), 123.4 (H-5), 121.9 (H-6), 130.1 (H-7), 114.1(H-8), 139.0 (H-9), 122.4 (H-10), 24.3 (H-1′), 121.5 (H-2′), 132.5 (H-3′), 25.7 (Me-3′cis), 19.0 (Me-3′trans), 29.8 (N-Me), 61.8 (OMe-4) [17].

Compound (**4**), ^1^H NMR (400 MHz, CD_3_OD): 7.58 s (H-1), 8.31 s (H-4), 9.71 s (H-6), 7.99 d (*J* = 9.0 Hz, H-9), 8.67 d (*J =* 9.0 Hz, H-10), 8.67 d (*J =* 9.0 Hz, H-11), 8.24 d (*J =* 9.0 Hz, H-12), 4.28 3H s (OMe-7), 5.51 3H s (N-Me), 6.29 2H s (-OCH_2_O-) [18].

Compound (**5**) ^1^H NMR (400 MHz, CDCl_3_): 7.32 s (H-1), 8.02 s (H-4), 10.72 s (H-6), 7.85 d (*J* = 9.0 Hz, H-9), 8.34 d (*J* = 9.0 Hz, H-10), 8.32 d (*J* = 9.0 Hz, H-11), 7.98 d (*J* = 9.0 Hz, H-12), 4.41 3H s (OMe-7), 4.04 3H s (OMe-8), 5.27 3H s (N-Me), 6.18 2H s (-OCH_2_O-) [19], [20].

Compound (**6**), ^1^H NMR (400 MHz, CDCl_3_): 7.82 s (H-1), 8.04 s (H-4), 10.72 s (H-6), 8.42 s (H-10, 8.23 d (*J =* 9.0 Hz, H-11), 7.85 d (*J =* 9.0 Hz, H-12), 4.41 3H s (OMe-7), 4.04 3H s (OMe-8), 3.88 3H s (OMe-9), 5.24 3H s (N-Me), 6.22 2H s (-OCH_2_O-) [19], [20].

Compound (**7**), ^1^H NMR (400 MHz, CDCl_3_): 8.20 d (J = 5.9 Hz, H-1), 8.73 d (*J* = 5.9 Hz, H-2), 8.49 d (*J* = 10 Hz, H-4), 7.09 d (*J* = 10 Hz, H-5), 8.17 d (*J* = 8.0 Hz, H-8), 7.59 dd (*J =* 8.0, 7.5 Hz, H-9), 7.81 dd (*J* = 8.4, 7.5 Hz, H-10), 8.65 d (*J =* 8.4 Hz, H-11) [21].

Compound (**8**), ^1^H NMR (400 MHz, CDCl_3_): 6.97 d (*J =* 1.2 Hz, H-2), 6.71 d (*J* = 7.0, 9.0 Hz, H-5), 6.76 dd (*J* = 8.0,2.0 Hz, H-6), 4.66 d (*J =* 5.2 Hz, H-7), 3.09 m (H-8), 4.25 dd (*J =* 9.6, 6.0 Hz, H-9α), 3.94 m (H-9β), 6.83br s (H-2′), 6.74 d (*J =* 8.8 Hz, H-5′), 6.76 d (*J =* 8.0 Hz, H-6′), 4.44 d (*J =* 4 Hz, H-7′), 3.09 m (H-8′), 4.25 dd (*J* = 9.6, 6.0 Hz, H-9′α), 3.94 m (H-9′β), 3.80 3H s (OMe-3), 5.88 2H s (3′,4′-OCH_2_O-), 4.50 (*J* = 9.6 Hz, H-1″), 5.44 (*J* = 6 Hz, H-2″), 1.69 3H s (Me-3″cis), 1.65 3H s (Me-3″trans). ^13^C (100 MHz, CDCl_3_): 135.1 (C-1), 106.5 (C-2), 147.9 (C-3), 148.0 (C-4), 112.9 (C-5), 119.4 (C-6), 85.8 (C-7), 54.4 (C-8), 71.7 (C-9), 133.5 (C-1′), 109.4 (C-2′), 147.1 (C-3′), 149.8 (C-4′), 108.2 (C-5′), 118.2 (C-6′), 85.9 (C-7′), 54.1 (C-8′), 71.7 (C-9′), 56.0 (OMe-3), 101.1 (3′,4′-OCH2O-), 65.8 (C-1″), 120.0 (C-2″), 137.6 (C-3″), 25.8 (Me-3″cis), 18.2 (Me-3″trans).

Compound (**9**), ^1^H NMR (400 MHz, CDCl_3_): 3.55 m(H-3), 5.37 d (*J* = 5.2 Hz, H-6), 0.72 s (H-18), 1.03 s(H-19), 1.04 d (*J* = 7.5 Hz, H-21), 5.18 dd (*J* = 15.2, 8.6 Hz, H-22), 5.04 dd (*J* = 15.2, 8.6 Hz, H-23), 0.83 d (*J* = 7.0 Hz, H-26), 0.88 d (*J* = 6.3 Hz, H-27), 0.83 t (*J* = 7.0 Hz, H-29) [22].

Compound (**10**), ^1^H NMR (400 MHz, CDCl_3_): 3.55 m (H-3), 5.37 d (*J* = 5.2 Hz, H-6), 0.70 s (H-18), 1.03 s (H-19), 0.95 d (*J* = 6.4 Hz, H-21), 0.84 d (*J* = 7.2 Hz, H-26), 0.86 d (*J* = 7.2 Hz, H-27), 0.87 t (*J* = 7.2 Hz, H-29) [22].

Compound (**11**), ^1^H NMR (400 MHz, CDCl_3_): 2.32 t (2H, *J* = 7.4 Hz, H-2), 1.63 m (2H, H-3), 1.27 m (20H, H-4 to 7, H-12 to 17), 2.03 m (4H, H-8, H-11), 5.36 m (2H, H-9, H-10), 0.88 t (3H, *J* = 6.8 Hz, Me-18), 3.68 s (3H, -OMe) [23].

Compound (**12**), ^1^H NMR (400 MHz, CDCl_3_): 2.32 t (2H, *J* = 7.4Hz, H-2), 1.63 m (2H, H-3), 1.27 m (28H, H-4 to 17), 0.88 t (3H, *J* = 6.8 Hz, Me-18), 3.68 s (3H, -OMe) [23].

Compound (**13**), ^1^H NMR (400 MHz, CDCl_3_): 2.32 t (2H, *J* = 7.4Hz, H-2), 1.63 m (2H, H-3), 1.27 m (28H, H-4 to 17), 0.88 t (3H, *J* = 6.8 Hz, Me-18) [23].

### 2.2. Molecular Docking with MPro

**Figure 3:**
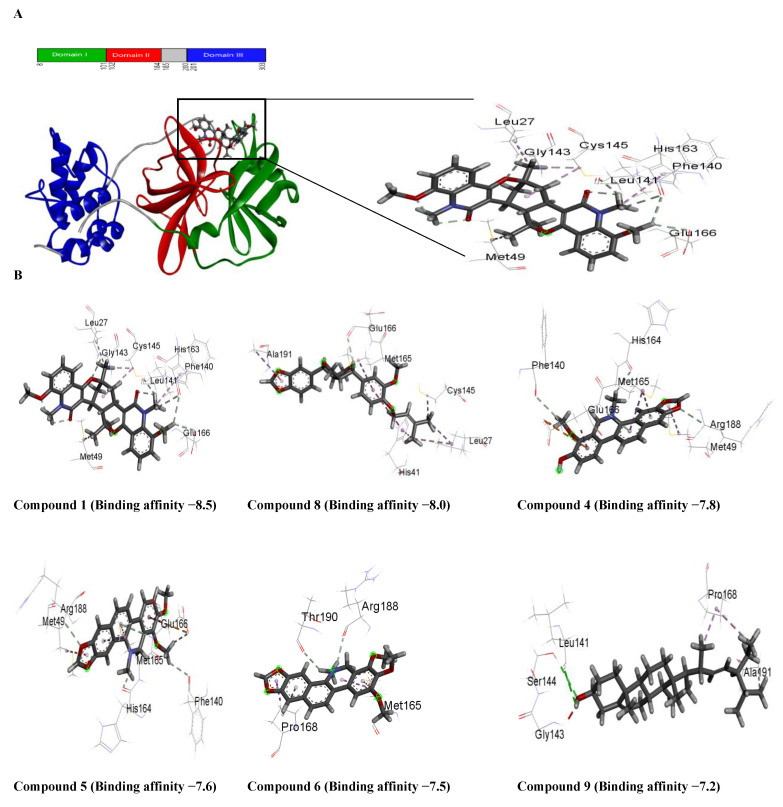
(**A**) Schematic 1D amino acid sequence of main protease and interaction of main protease with Compound **1**. (**B**) Interaction of main protease with different Compound and binding affinity.

Molecular docking was performed on thirteen compounds to study their interactions with the Mpro (Table 3; Figure 3) using AutoDock Vina. The highest negative binding affinity is observed for compound **1,** indicating that it can potentially serve as a chemical scaffold to develop a potent Mpro inhibitor.

#### 2.2.1. Molecular Interaction of the Four Compounds with Mpro

**Figure 4:**
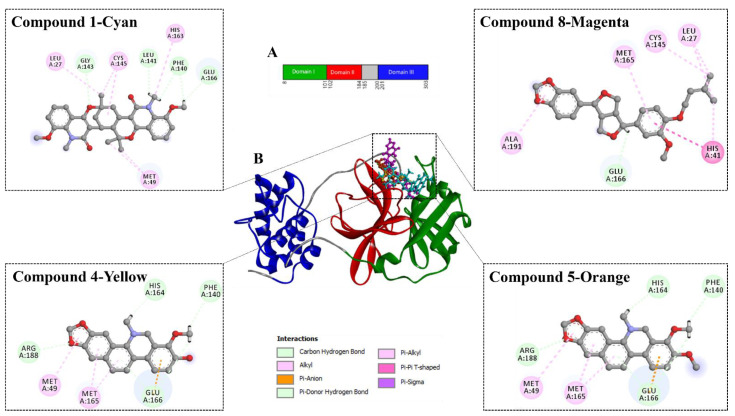
Domain organization and interaction of Mpro with 4 compounds. (**A**) The interdomain borders are labeled with amino acid residue numbers. (**B**) Three-dimensional representation of Mpro (Domain I—Green; Domain II—Red; Domain III—Blue) interacting with 4 compounds. Here Compound **1**—Cyan; Compound **8**—Magenta; Compound **4**—Yellow; Compound **5**—Orange.

**Figure 5:**
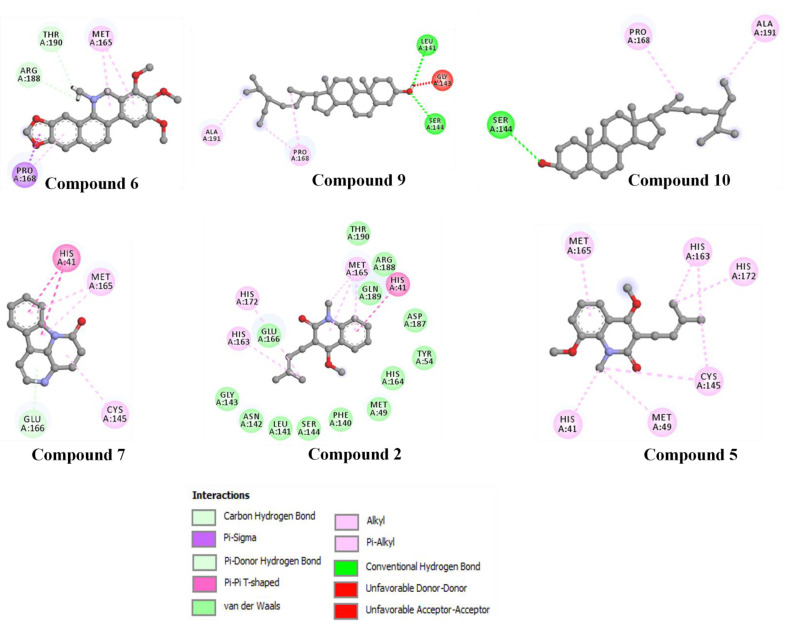
Non-bonding interactions of different compounds with Mpro (Pose predicted by AutoDock Vina).

Non-covalent interactions are known to play a major role, as they are considered to drive protein–ligand interactions [24]. For example, hydrogen bond and hydrophobic interactions were found to be involved in the binding of the compounds with Mpro when the poses were estimated with AutoDock Vina (Figure 3 and Figure 4). Among the thirteen compounds, the highest negative binding affinity was observed for compound **1** (−8.5 kcal/mol). Compound **1** formed five hydrogen bonds and six hydrophobic bonds with Mpro. In the compound **8**-Mpro complex, the complex was stabilized by one hydrogen bond and seven hydrophobic bonds. The compound **4**-Mpro complex formed a stable network with five hydrogen bonds, four hydrophobic bonds, and one electrostatic bond, whereas in the compound **5**-Mpro complex, five hydrogen bonds, four hydrophobic bonds, and one electrostatic bond were detected (Figure 4). Similar interactions were shown by the other eight compounds with Mpro (Table 3 and Figure 5).

#### 2.2.2. Result Obtained from Molecular Dynamics Simulation

**Figure 6:**
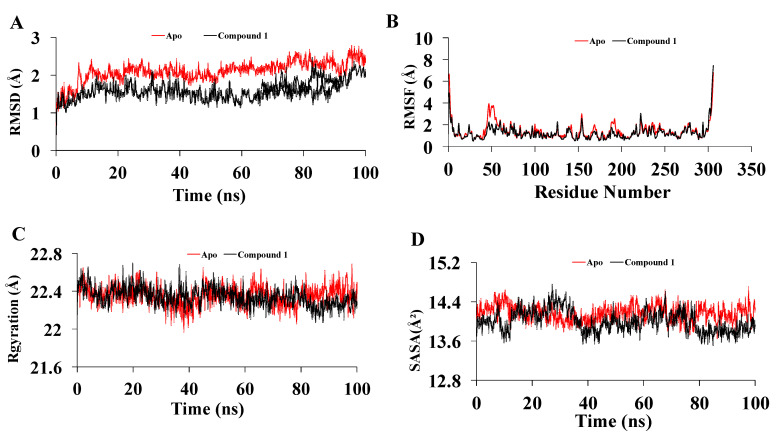
(**A**) Root means square deviation values of C-α atom of Mpro in the complexes with compound **1** and apo form along the 100 ns MD simulations. The structural changes of Mpro by means of (**B**) root means square fluctuations, (**C**) solvent accessible surface area, and (**D**) radius of gyration formed during the simulation.

For the promising Mpro inhibitors, we conducted molecular dynamics and explored the MD stability of each MD system. A 100 ns MD simulation for the complex of Mpro with new compound **1** was performed for 100 ns. The Apo form of the Mpro was also employed for the MD run. Compound 1 has lower RMSDs (0.4–2.4) for the alpha-carbon atoms than apo form (0.4–2.7), implying that compound **1** may be more stable in physiological conditions. In Figure 6A, RMSD values of compound **1** were slightly increased to 2.1 Å after 22 ns and showed a few smaller fluctuations at 26, 74, and 82 ns. Apart from this, the trajectories generated throughout the whole run were stable. Thus, higher structural stability in the Mpro-compound **1** complex was identified. On the other hand, the RMSD of Apo exhibited similar higher fluctuations over the whole run. The average RMSD for compound 1-Mpro was lower (1.6 Å) compared to apo-Mpro (2.1 Å).

The Root mean square fluctuations (RMSF) property is widely used to capture protein dynamics conservation [25]. The highest degree of flexibility was detected for apo-Mpro, whereas a similar fluctuation (average 1.2 Å) was noticed for compound 1-Mpro. The lower fluctuation indicated a higher protein structural stability. In addition, the visual analysis of MD simulation trajectories showed that compound 1 was involved in major binding interactions with the hotspot residues (HIS163, GLU166, CYS145, GLY143, HIS172, PHE140, HIS41, THR25, MET49, MET165, and GLN189) of the Mpro protein (Figure 6B).

The radius of gyration is a parameter that indicates protein structural compactness. During the simulation run, a lower degree of fluctuation with its consistency suggested the greater compactness and rigidity of the system [25]. Throughout the whole run, a similar radius of gyration was identified for compound **1**-Mpro while comparing with apo-Mpro. The average radius of gyration of apo-Mpro was nearly same as the compound **1**-Mpro (Figure 6C).

**Figure 7:**
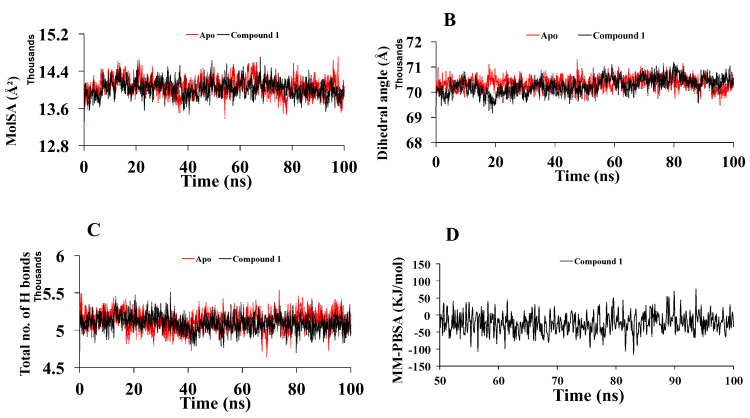
(**A**) Molecular surface area value of apo-Mpro and compound **1-Mpro** along the 100 ns MD simulations. The structural changes of Mpro by means of (**B**) dihedral angle, (**C**) total number of H bonds formed during the simulation, and (**D**) binding free energy analysis of Compound **1**-Mpro.

The expansion of protein volume is indicated by a higher solvent-accessible surface area (SASA) value, and a low fluctuation is anticipated all through the simulation time. The assessment of SASA indicated the highest value for apo-Mpro (average of 14,160 Å^2^) compared tothe compound **1**-Mpro complex (average of 14,000 Å^2^) (Figure 6D). The molecular surface area (MolSA) of the drug–protein complexes was also determined. In this exploration, Compound **1** revealed similar MolSA (average 14,027 Å^2^) as apo-Mpro (Figure 7A). A noticeable difference was detected for dihedral angles of compound 1-Mpro (average 70,261) (Figure 7B) compared to apo-Mpro. Hydrogen bonds play a vital role in molecular recognition and the stability of the whole protein structure. During the whole 100 ns, the formed intermolecular hydrogen bonds are composed of the trajectories. Compound **1**-Mpro showed the comparable number of hydrogen bonds (Figure 7C).

#### 2.2.3. Binding Free Energy Analysis

To understand how the structural changes influence ligand binding, each snapshot was subjected to MM-PBSA calculation. The binding free energy of each snapshot containing a protein–ligand complex is shown in Figure 7D. The average binding free energy of compound **1** was calculated as −23.74 KJ/mol. It showed greater free energy of binding at 89.9 ns and 93.5 ns, which was calculated as 69.53 KJ/mol and 74.73 KJ/mol, respectively.

## 3. Discussion

Compound **1** indicated the highest binding affinity of −8.5 kcal/mol. When docking results are considered with the other three compounds, together with hydrogen bond and hydrophobic bonds, compound **1** shows very strong binding interactions with the important amino acids of Mpro [26,27]. Compared to the others, through hydrogen bond and alkyl interactions, it also showed better and more appropriate interactions with the significant residues. It can promote more interactions than the others, contributing to a higher binding affinity. Amino acid residues, MET49, CYS145, GLU166, HIS163, and MET165 in the catalytic domain of Mpro were found to participate in non-covalent interactions with most of the compounds (Figure 4 and Table 3). By MD simulation, compound **1** showed a lower average RMSD values (1.6) than apo-Mpro, indicating more structural stability with Mpro in bound states. From the RMSF values, it was observed that compound **1** had more acceptable binding stability with Mpro (Figure 6B), which demonstrates important interaction with significant residues and their compactness in a complex form with Mpro. The compound **1** was found to be stable (Figure 6C,D), as revealed by the Rg and SASA values. Furthermore, the compound **1**-Mpro complex showed a comparable result for MolSA (Figure 7A). All analyses from the MD simulations support the docking results, signifying that our isolated compound **1** (2,11-didemethoxy-vepridimerine A) has potential to inhibit the activity of Mpro of SARS-CoV-2.

## 4. Materials and Methods

### 4.1. General Experimental Procedures

In the present work, NMR spectra were measured at 400 MHz for ^1^H NMR spectra and 100 MHz for ^13^C on a Bruker 400^TM^ ASCEND spectrometers (Bruker UK, Coventry, United Kingdom). The sample was dissolved in CDCl_3_-d. Chemical shifts (δ) are reported on a ppm using tetramethyl silane (TMS) and/or residual solvent peak as an internal reference. Coupling constants (*J*) are given in hertz. High resolution electrospray ionization mass spectrometry (HRESIMS) was used for obtaining the mass spectrum. Column chromatography (CC) was carried out using powdered silica gel 60 H (Kieselgel 60, mesh 70–230). The glass column, having a length of 92 cm and diameter 4.5 cm, was packed with silica gel. Thin layer chromatography (TLC) was obtained using pre-coated silica gel plates (silica gel 60 F254, aluminum sheets, E-Merck, Germany) and the established chromatogram was detected under UV light (366 nm and 254 nm). The glowing and quenched spots were marked, and the recognition was then visualized by spraying with Vanillin-H_2_SO_4_ and modified Dragendorff’s reagent. 

### 4.2. Plant Material

The root bark of *Zanthoxylum rhetsa* was collected from Dhaka, Narsingdi district of Bangladesh in August 2013. The plants were identified by an expert at the Bangladesh National Herbarium and where a voucher specimen was deposited (DACB accession number is 42,528). The plant parts were cleaned properly. The parts were cut into small pieces and subjected to shade drying for a week. The dried plant part was then crushed into coarse powder by a high-capacity grinding machine with proper care.

### 4.3. Extraction and Isolation

The powdered root bark of *Zanthoxylum rhetsa* (3.5 kg) was soaked in MeOH (5 L) at room temperature for two weeks. The MeOH extracts were combined and evaporated in a vacuum to yield a dark brown residue (40 g, semi-dry). The crude extract was then fractionated sequentially with petroleum ether (9 g, PE), ethyl acetate (5 g, EtOAc) and chloroform (12 g, CHCl_3_) by continuous stirring. The CHCl_3_ soluble fraction (12 g) was subjected to column chromatography (CC) over silica gel [PE-CH_2_Cl_2_ gradient (100:0–0:100, *v*/*v*) to CH_2_Cl_2_-EtOAc (99:1–0:100, *v*/*v*) to EtOAc-MeOH (99:1–0:100, *v*/*v*)] to afford 700 fractions each with 20 mL solution. Based on TLC screening, fractions 34 to 40 (EtOAc-CH_2_Cl_2_, 32:68, *v*/*v*), 41 to 45 (EtOAc-CH_2_Cl_2_, 34:66, *v*/*v*), 46 to 56 (EtOAc-CH_2_Cl_2_, 34:66–36:64, *v*/*v*), 60 to 62 (EtOAc-CH_2_Cl_2_, 36:64, *v*/*v*), 89 to 90 (EtOAc-CH_2_Cl_2_, 44:56, *v*/*v*) and 175 to 176 (MeOH-EtOAc, 15:85–18:82, *v*/*v*) were combined, giving six subfractions (Fr.1–Fr.6). All the subfractions were further fractionated by gel permeation chromatography with sephadex LH-20, using petroleum ether/chloroform (20:80, *v*:*v*) to afford 1 to 20 subfractions, containing 1.5 mL each. After TLC analysis, subfractions Fr.3.7–Fr.3.10 are mixed together followed by TLC eluting with toluene/EtOAc (90:10) to come up compound **1** (9 mg, R_f_ = 0.40), the sample containing TLC plate was run four times in the solvent system. Concentrated subfractions Fr.5.4–5.5 gave crystals and after recrystallization afforded found compound **7** (5 mg, R_f_ = 0.37). Subjected to TLC of subfraction Fr.6.3–Fr.6.6 eluting with toluene/EtOAc (85:15, *v*/*v*) to afford mixture of compound **5** (4.5 mg, R_f_ = 0.49) and compound **6** (9 mg, R_f_ = 0.49). 

The entire pet ether soluble fraction (9 g) was exposed to column chromatography (CC) by means of silica gel by gradient elution with pet ether/dichloromethane/ethyl acetate/methanol [PE-CH_2_Cl_2_ gradient (100:0–0:100, *v*/*v*) to CH_2_Cl_2_-EtOAc (99:1–0:100, *v*/*v*) to EtOAc-MeOH (99:1–0:100, *v*/*v*)] to afford 551 fractions each with 20 mL. The fractions 1 to 19 (Pet ether,100:0, *v*/*v*), the fractions 60 to 99 (CH_2_Cl_2_ in pet ether, 5:95–15:85, *v*/*v*),140 to 175 (CH_2_Cl_2_ in pet ether, 50:50–100:0), 219 to 279 (EtOAc in CH_2_Cl_2_, 15:85–25:75, *v*/*v*), 340 to 360 (EtOAc in CH_2_Cl_2_, 30:70–75:25, *v*/*v*), 370–410 (EtOAc in CH_2_Cl_2_, 80:70–100:0, *v*/*v*) and 480 to 551 (EtOAc in MeOH, 44:56, *v*/*v*) were mixed together affording seven fractions (Fr.1–Fr.7) based on TLC analysis, respectively. Fr.5 and Fr.7 were resubmitted to Sephadex LH-20 eluted with petroleum ether/chloroform (20:80, *v*:*v*) and afforded another twenty sub fractions of each fraction. Fr.5.7–Fr.5.14 mixed together were subjected to TLC run with toluene/EtOAc (97:3, *v*/*v*) for three times to yield compound **2** (8.7 mg, R_f_ = 0.80) and compound **3** (8.7 mg, R_f_ = 0.80). Subfractions Fr.4.7–Fr.4.15 were added together, followed by and subjected to TLC eluted with toluene/EtOAc (97:3) to yield compound **8** (4.5 mg, R_f_ = 0.49). From Fr. 2 and Fr. 3 get compound **9** (20 mg, R_f_ = 0.55) and compound **10** (20 mg, R_f_ = 0.55). Fr.1 afforded compound **11** (8 mg, R_f_ = 0.46), compound **12** (8 mg, R_f_ = 0.47) and compound **13** (8 mg, R_f_ = 0.46) by TLC eluting with toluene/EtOAc (95.5:0.5, *v*:*v*).

### 4.4. Methodologies of Molecular Docking on SARS-CoV-2 Main Proteases

#### 4.4.1. Molecular Docking

Optimization at the semi-empirical PM6 method (gaseous phase) of the structure of the selected compounds was employed [28]. Later, the vibrational frequencies were computed and the absence of imaginary frequencies confirmed the stationary points correspond to minima on the Potential Energy Surface. From the RCSB Protein Data Bank (PDB ID: 6LU7) the crystal structure of the Mpro was taken. After that it was energy-minimized through a Swiss PDB Viewer [29]. The BIOVIA discovery studio (version 4.5, Vélizy-Villacoublay, France) software package was used to eliminate all of the hetero atoms and water molecules present in the crystal structure [30]. Molecular docking between Mpro with thirteen compounds was performed by AutoDock Vina protocol for the potential interactions and activity predictions [31]. The Mpro active site was set around in the docking grid box, where the center was around X = −9.83, Y = 14.38, Z = 68.48 and the dimensions were X: 24.69, Y: 31.64, and Z: 29.97. Finally, for detecting the non-covalent interaction in the docked drug–protein complex, BIOVIA discovery studio (version 4.5, Vélizy-Villacoublay, France), were visualized and detected.

#### 4.4.2. Molecular Dynamics

Along with the apo form of Mpro, MD simulations of new compound **1** was performed using the YASARA software package, 21.8.27 (Vienna Austria). These simulations were executed to study the virtual stability of the ligand locate in the binding pocket. A dynamics system consisted of one copy of Mpro, one copy of docked compound, water molecules, and 0.9% NaCl at 298K temperature. The entire system was neutralized. The MD system was first relaxed for a protein–ligand complex via a series of procedures such as the steepest descent minimization and simulated annealing minimization of the solvent. For describing the macromolecular system, the AMBER14 force field was utilized. Three-dimensional periodic boundary conditions (PBC) were applied wherever the cell size was 20 Å larger than the Mpro size in all cases. Throughout all simulations, the Particle-Mesh-Ewald (PME) method was employed for long-range electrostatic interactions (with a cut-off of 8 Å). The simulation temperature was controlled by the Berendsen thermostat process. For performing 100 ns MD simulation, a time step of 1.25 fs was maintained. The snapshots were taken at every 100 ps. Depending on previous MD data analysis process root-mean-square deviation (RMSD), root-mean-square fluctuation (RMSF), radius of gyration (Rg), and solvent accessible surface area (SASA), molecular surface area (MolSA), dihedral angle, and total number of hydrogen bonds data were retrieved from the MD simulations [32,33,34]. At that point, 100 snapshot conformers were generated and saved in PDB format at every 1 ns of 100 ns MD simulation.

#### 4.4.3. Binding ENERGY Calculation MM-PBSA

By using YASARA software, Gibbs free energy was calculated for analyzing the binding affinity of ligands. Interaction energy for Mpro with compound **1** was calculated. MM-PBSA methods were implemented for 51 to 100 ns.

## 5. Conclusions

In this study we identified a new natural compound, 2,11-didemethoxy-vepridimerine A (**1**), the dimeric prenylated quinolone alkaloid, that showed promising interaction with SARS-CoV-2 MPro. Mpro, coronavirus’ main protease, is a critical enzyme mediating the production of CoV functional proteins. The molecular docking results were supported by the MD simulation calculations. This suggests that the isolated compound 1 can potentially inhibit the activity of Mpro, and can be used as a lead compound for an Mpro targeting drug-discovery program. However, these results need to be validated using in vitro wet lab experiments to fully understand the potential utility of this chemical class.

## Figures and Tables

**Table 1:** NMR spectral data (400 MHz, CDCl_3_) for compound **1**.

Position	δ_H_ ^a^	δ_C_ ^b^	HSQC	HMBC
1	---	148.5		
2	7.05 dd (*J* = 8.0, 1.3 Hz)	114.1	114.1	130.6 (C-18a), 116.0 (C-4)
3	7.17 dd (*J* = 8.0, 7.9 Hz)	122.0	122.0	148.5 (C-1), 118.3 (C-4a)
4	7.63 dd (*J* = 7.9, 1.3 Hz)	116.0	116.0	c
4a	---	118.3		
4b	---	158.1		
6	---	80.0		
6a	2.23 d (*J* = 6.3 Hz)	43.5	43.5	c
7	3.70 br s	27.8	27.8	c
7a	---	108.3		
8	---	163.0		
9a	---	130.4		
10	---	148.3		
11	7.04 dd (*J* = 8.0, 1.3 Hz)	113.7	113.7	116.2 (C-13)
12	7.14 dd (*J* = 8.0, 7.9 Hz)	122.3	122.3	148.3 (C-10), 118.8 (C-13a)
13	7.73 dd (*J* = 7.9, 1.3 Hz)	116.2	116.2	
13a	---	118.8		
13b	---	154.9		
15	---	77.1		
16	1.57 dd (*J* = 14.2, 14.2 Hz) 3.20 ddd (*J* = 14.2, 5.5, 2.4 Hz)	39.5	39.5	
16a	3.05 ddd (*J* = 12.5, 6.0, 5.7 Hz)	26.5	26.5	c
16b	---	109.7		
17	---	163.9		
18a	---	130.6		
19	1.74 ddd (*J* = 13.8, 2.4, 2.4 Hz) 2.19 dd (*J* = 13.8, 2.8 Hz)	32.2	32.2	c
Me-6	1.55, 3H s	24.9	24.9	43.5 (C-6a), 29.2 (Me-6)
Me-6	1.87, 3H s	29.2	29.2	80.0(C-6), 43.5 (C-6a), 24.9 (Me-6)
Me-15	1.55, 3H s	28.7	28.7	77.1 (C-16), 39.5 (C-15), 32.2 (C-19)
OMe-1	3.89, 3H s	56.9	56.9	56.9 (C-1)
OMe-10	3.88, 3H s	56.7	56.7	56.7 (C-10)
NMe-9	3.93, 3H s	35.2	35.2	130.4 (C-9a), 163.0 (C-8)
NMe-18	3.88, 3H s	34.8	34.8	130.6 (C-18a), 163.9 (C-17)

a = measured in 400 MHz, b = measured in 100 MHz, δ are reported on a ppm.

**Table 2:** Comparison with the signals (H_a_-H_g_ and ^13^C NMR) of the previously known vepridimerine A and compound **1**.

	Compound 1		Vepridimerine A [16]	
Position	δ_H_ ^a^	δ_C_ ^b^	δ_H_	δ_C_
H_a_	1.74 ddd (*J* = 13.8, 2.4, 2.4 Hz)	32.2	1.70 ddd (*J* = 13.7, 2.8, 2.2 Hz)	32.2
H_b_	2.19 dd (*J* = 13.8, 2.8 Hz)	32.2	2.14 dd (*J* = 13.7, 2.7 Hz)	32.2
H_c_	3.70 br s	27.8	3.61 ddd (*J* = 2.8, 2.7, 1.0 Hz)	26.2
H_d_	2.23 d (*J* = 6.3 Hz)	43.5	2.16 dd (*J* = 6.1, 1.0 Hz)	43.6
H_e_	3.05 ddd (*J* = 12.5, 6.0, 5.7 Hz)	26.5	2.96 ddd (*J* = 13.4, 6.1, 5.4 Hz)	27.6
H_f_	3.20 ddd (*J* = 14.2, 5.5, 2.4 Hz)	39.5	3.10 ddd (*J* = 14.2, 5.4, 2.2 Hz)	39.5
H_g_	1.57 dd (*J* = 14.2, 14.2 Hz)	39.5	1.56 dd (*J* = 14.2, 13.4 Hz)	39.5

a = measured in 400 MHz, b = measured in 100 MHz, δ are reported on a ppm.

**Table 3:** Non-covalent interactions of isolated compounds with Mpro (PDB ID 6LU7) (Pose predicted by AutoDock Vina).

Compound	Binding Affinity	Hydrogen Bond (AA…ligand)	Hydrophobic Interaction (AA…ligand)	Electrostatic Interaction (AA…ligand)
**1**		GLY143 (2.601) C-H…O-C)	CYS145 (4.789) Alkyl	
		LEU141 (2.530) C-O…H-C)	MET49 (4.276) Alkyl	
		PHE140 (2.975) C-O…H-C)	MET49 (4.353) Alkyl	
	−8.5	PHE140 (2.667) C-O…H-C	LEU27 (4.208) Alkyl	
		GLU166 (2.672) C-O…H-C	CYS145 (3.496) Alkyl	
			HIS163 (5.423) Pi-Alkyl	
**8**		GLU166 (2.615) C-O…H-C)	HIS41 (5.823) Pi-Pi T-shaped	
			LEU27 (4.72) Alkyl	
			LEU27 (4.383) Alkyl	
	−8.0		CYS145 (3.967) Alkyl	
			HIS41 (4.504) Pi-Alkyl	
			MET165 (5.479) Pi-Alkyl	
			ALA191 (5.124) Pi-Alkyl	
**4**		ARG188 (2.792) C-H…O-C	GLU166 (2.687) Pi-Sigma	GLU166 (4.933) Pi-Anion
		HIS164 (2.549) C-O…H-C	MET165 (4.778) Pi-Alkyl	
	−7.8	PHE140 (2.845) C-O…H-C	MET165 (4.741) Pi-Alkyl	
		GLU166 (2.613) C-O…H-C	MET49 (4.649) Pi-Alkyl	
		GLU166 (3.047) Pi-Donor		
**6**		ARG188 (2.784) C-H…O-C	GLU166 (2.745) Pi-Sigma	GLU166 (4.926) Pi-Anion
		HIS164 (2.509) C-O…H-C	MET165 (4.815) Pi-Alkyl	
	−7.6	PHE140 (2.724) C-O…H-C	MET165 (4.753) Pi-Alkyl	
		GLU166 (2.755) C-O…H-C	MET49 (4.658) Pi-Alkyl	
		GLU166 (3.041) Pi-Donor		
**5**			PRO168 (2.537) Pi-Sigma	
		ARG188 (2.480) C-O…H-C	MET165 (4.823) Alkyl	
	−7.5	THR190 (2.758) C-O…H-C	PRO168 (4.537) Pi-Alkyl	
			MET165 (5.086) Pi-Alkyl	
**9**		SER144 (2.737) O-H…O-C	ALA191 (3.775) Alkyl	
	−7.2	LEU141 (2.757) C-O…H-O	PRO168 (4.205) Alkyl	
			PRO168 (3.972) Alkyl	
**10**	−6.7	SER144 (2.710) C-H…O-C	ALA191 (4.156) Alkyl	
			PRO168 (4.156) Alkyl	
**2**	−6.3		HIS41 (4.917) Pi-Pi T-shaped	
			MET165 (4.480) Alkyl	
			HIS163 (4.690) Pi-Alkyl	
			HIS172 (5.434) Pi-Alkyl	
			MET165 (4.759) Pi-Alkyl	
			MET165 (5.048) Pi-Alkyl	
**3**	−6.1		CYS145 (4.795) Alkyl	
			MET49 (5.120) Alkyl	
			CYS145 (4.953) Alkyl	
			HIS41 (3.736) Pi-Alkyl	
			HIS163 (4.672) Pi-Alkyl	
			HIS163 (4.793) Pi-Alkyl	
			HIS172 (4.905) Pi-Alkyl	
			MET165 (4.428) Pi-Alkyl	
			MET165 (4.063) Alkyl	
			HIS41 (4.707) Pi-Alkyl	
			HIS41 (4.680) Pi-Alkyl	

## Data Availability

Not applicable.

## Supplementary Materials

The supporting information can be downloaded at: https://www.mdpi.com/article/10.3390/molecules27238191/s1. References [17,18,19,20,21,22,23] are cited in Supplementary Materials.

## Sample Availability

Not available.
