# Supplementary material for: Isolation and In Silico Prediction of Potential Drug-like Compounds with a New Dimeric Prenylated Quinolone Alkaloid from Zanthoxylum rhetsa (Roxb.) Root Extracts Targeted against SARS-CoV-2 (Mpro)
Source: Molecules. 2022 Nov 24;27(23):8191. doi: 10.3390/molecules27238191 (PMC9737416; doi:10.3390/molecules27238191)
Supplement: Supplementary file 1 [file molecules-27-08191-s001.zip › molecules-2001486-supplementary.pdf]

Supplementary Materials: NMR spectrum of compound 1 characterized as a new dimeric prenylated quinolone 2,11-didemethoxy-vepridimerine A

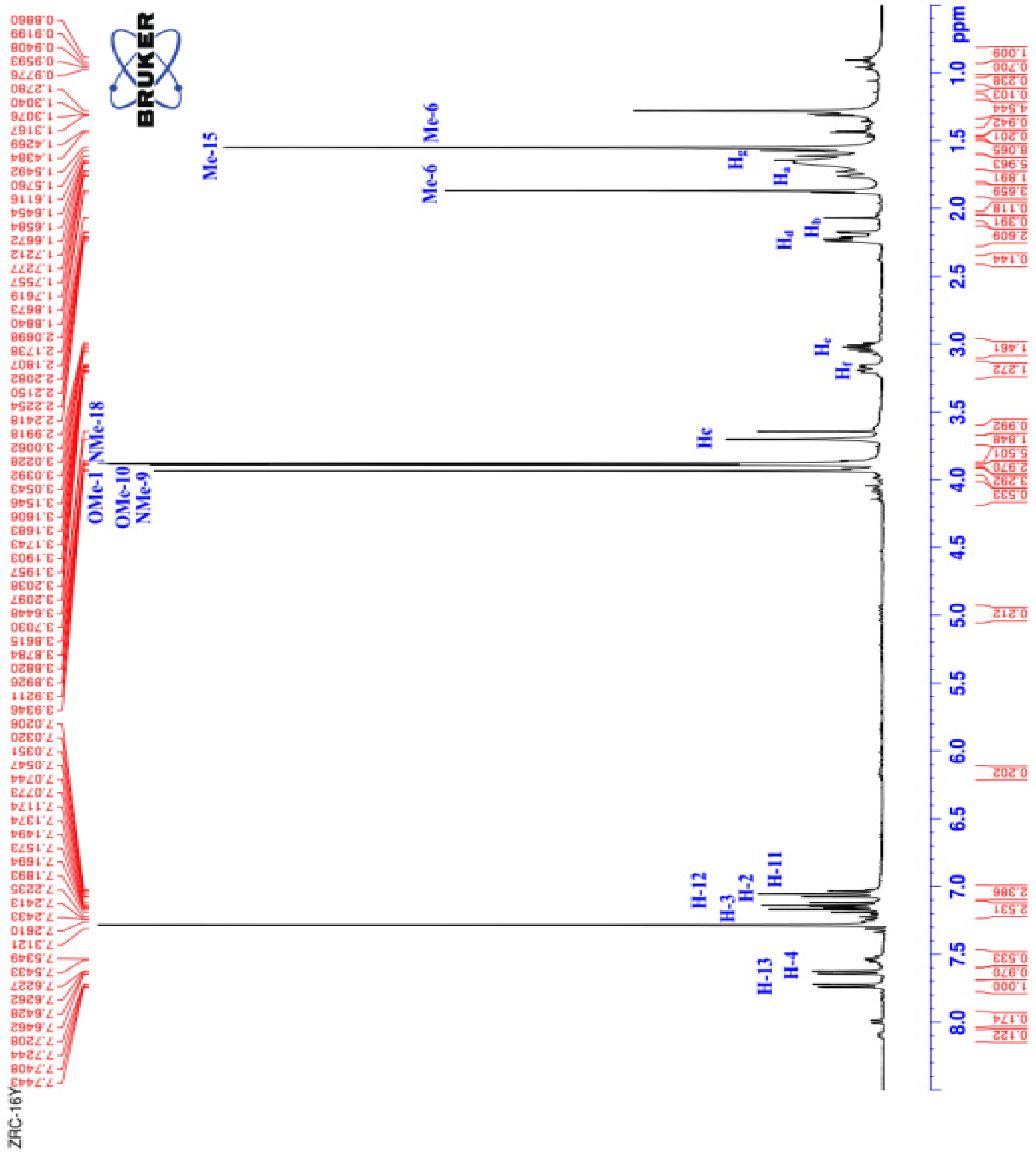

Figure S1. ^{1}H NMR (400 MHz, $CDCl_3$) spectrum of Compound **1** (ZRC-16Y)

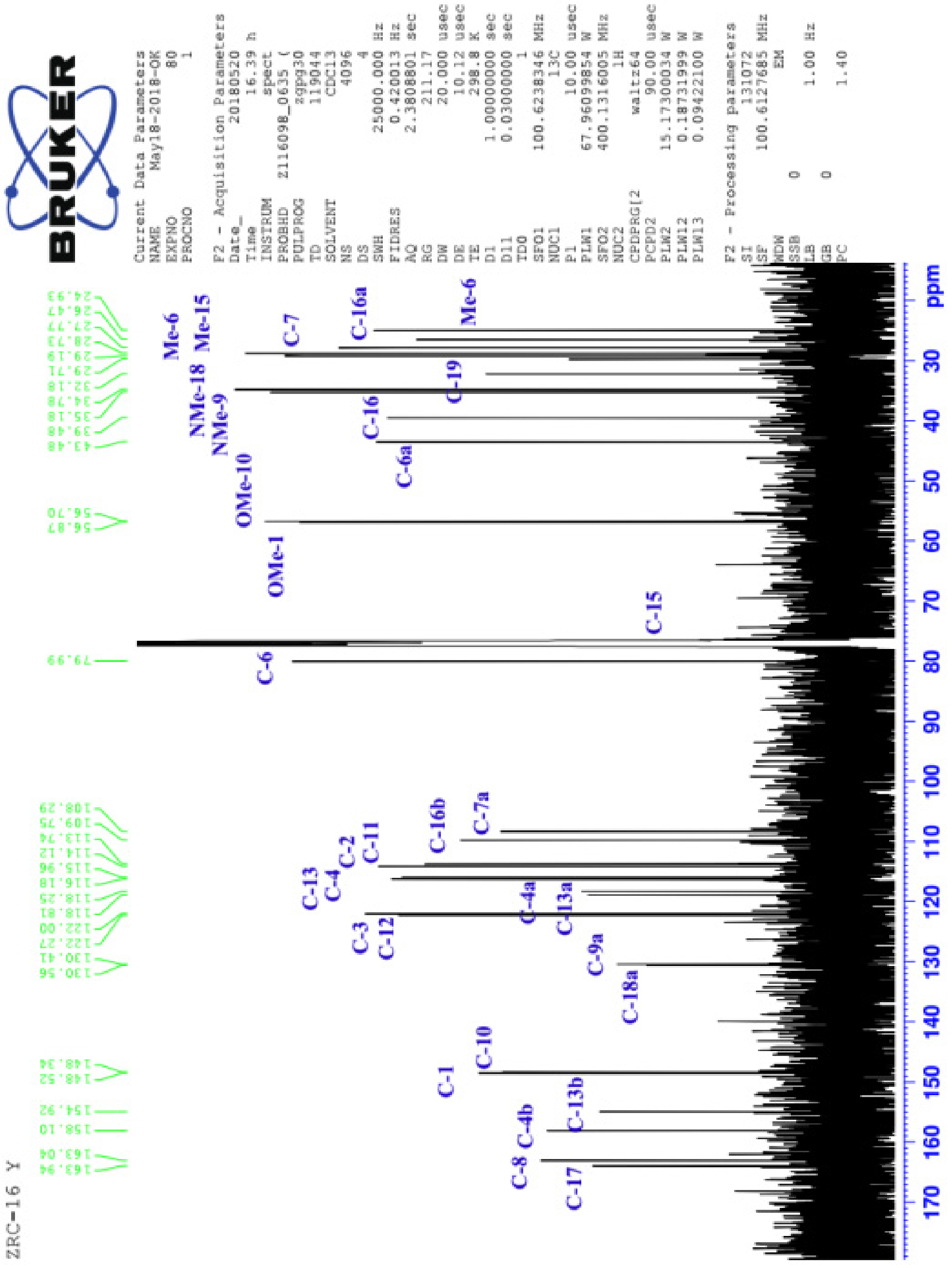

Figure S2. ^{13}C NMR (100 MHz, $CDCl_3$) spectrum of compound **1** (ZRC-16Y)

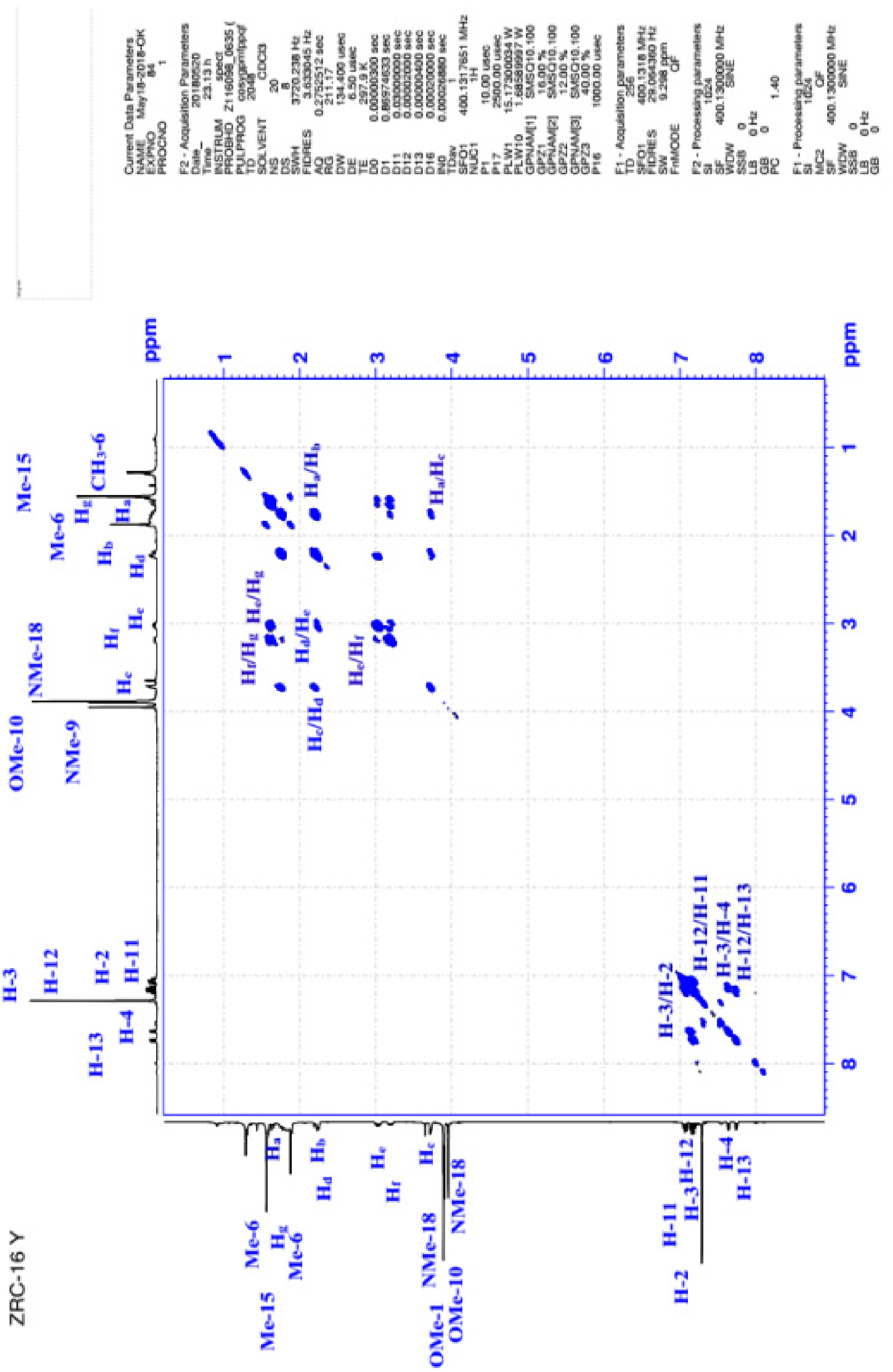

Figure S3. COSY (400 MHz, $CDCl_3$) spectrum of compound **1** (ZRC-16Y)

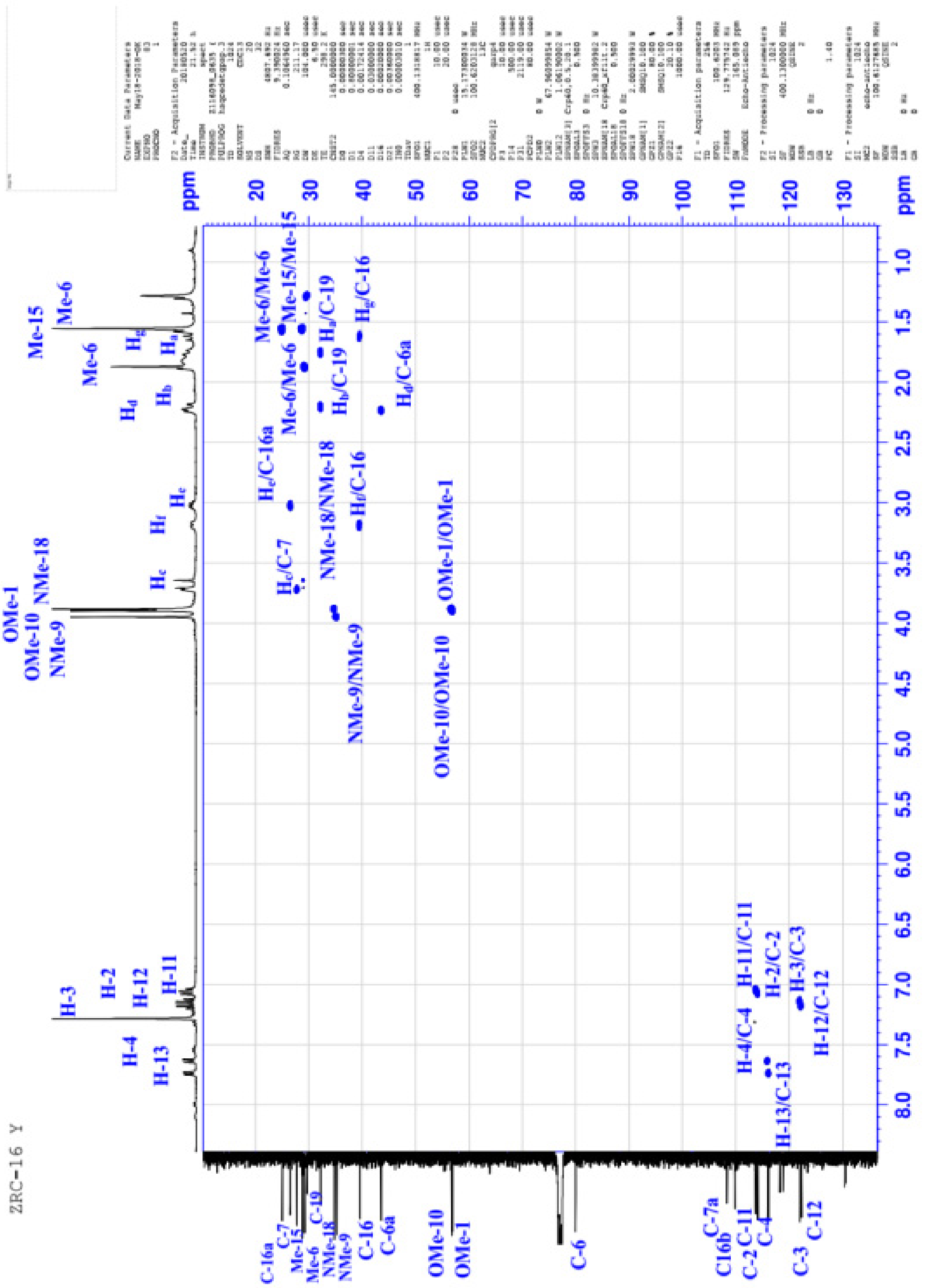

Figure S4. HSQC (400 MHz, $CDCl_3$) spectrum of compound **1** (ZRC-16Y)

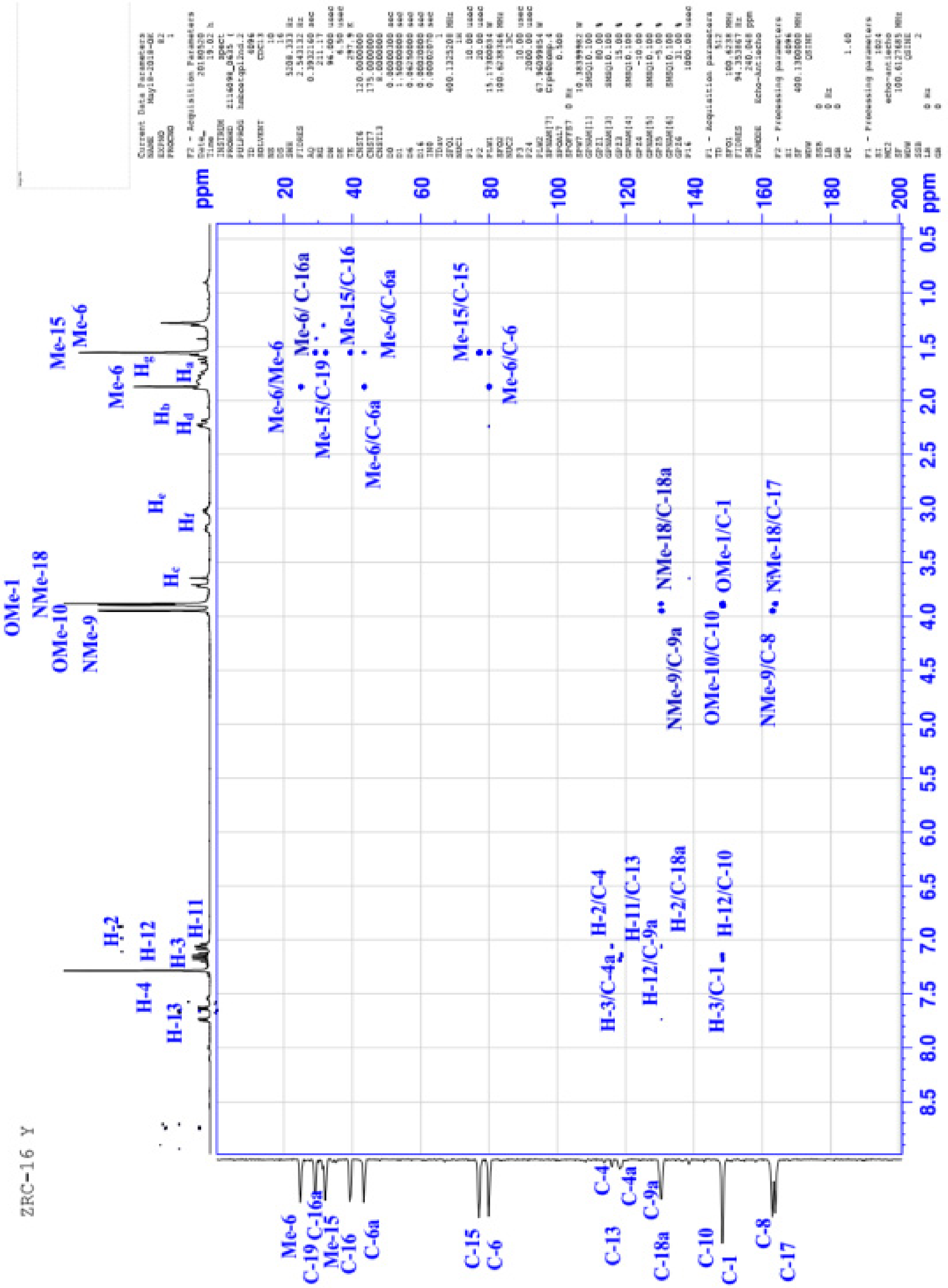

Figure S5. HMBC (400 MHz, $CDCl_3$) spectrum of compound **1** (ZRC-16Y)

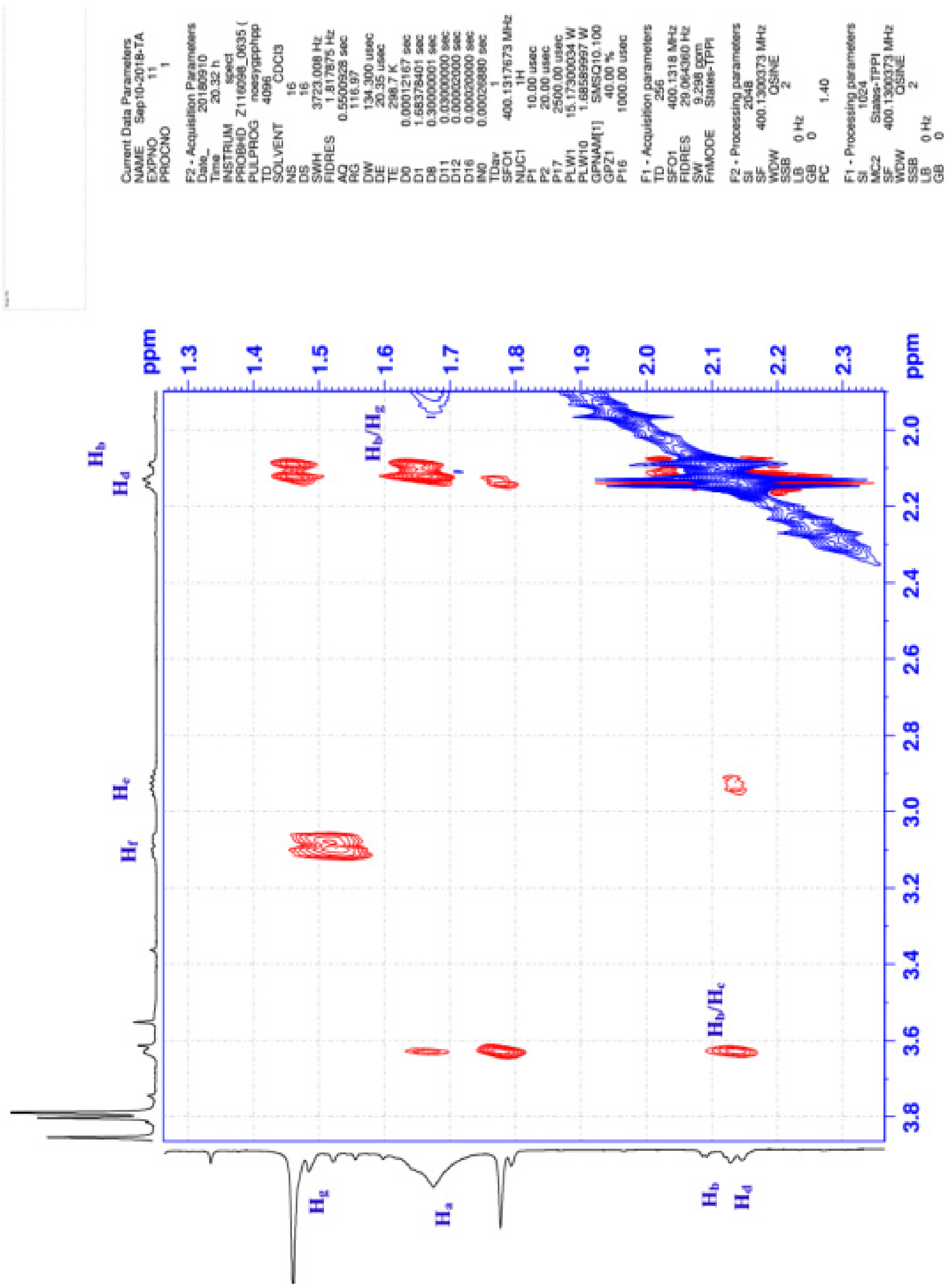

Figure S6. NOSEY (400 MHz, $CDCl_3$) spectrum of compound **1** (ZRC-16Y)

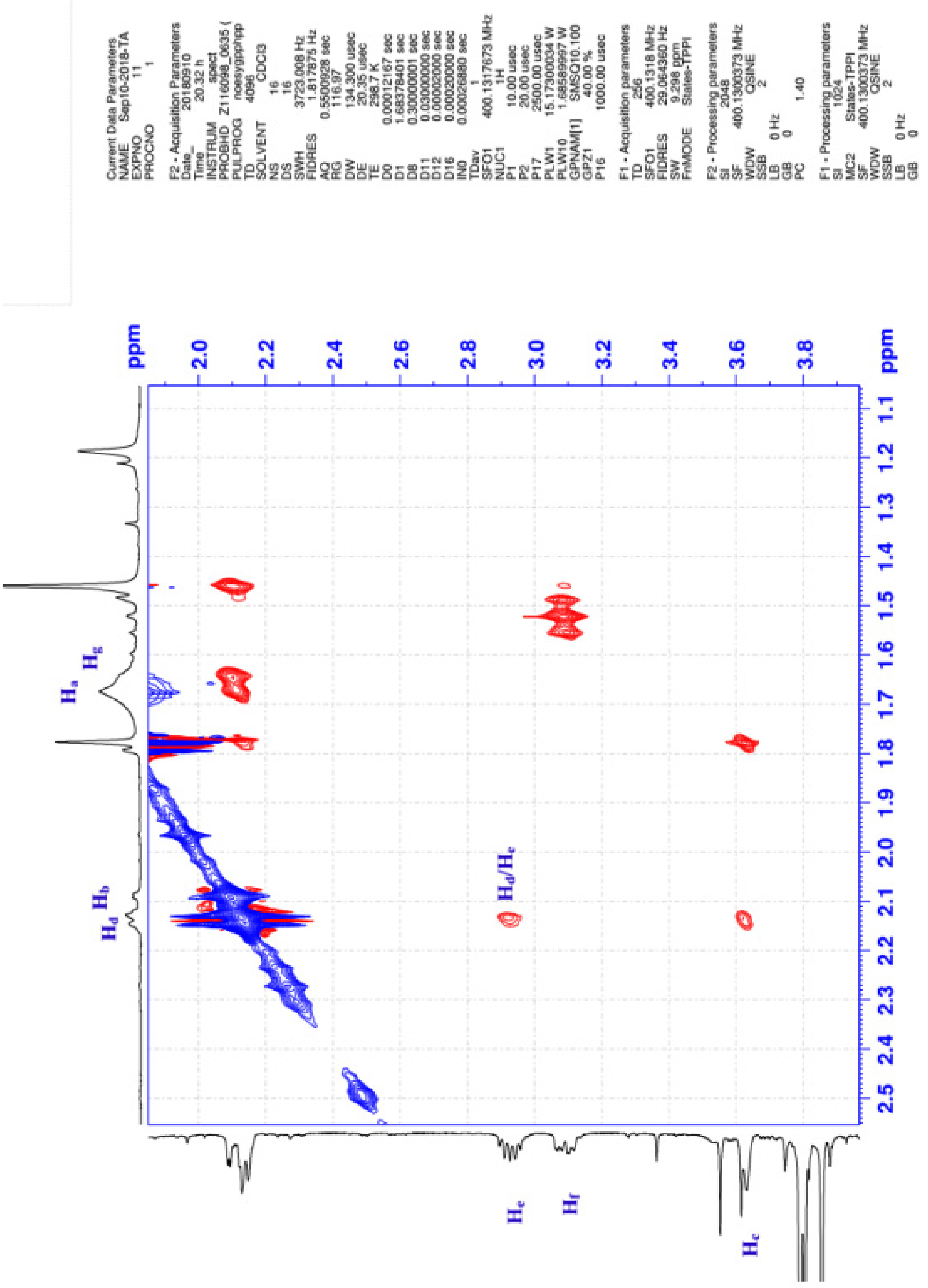

Figure S7. NOSEY (400 MHz, $CDCl_3$) spectrum of compound **1** (ZRC-16Y)

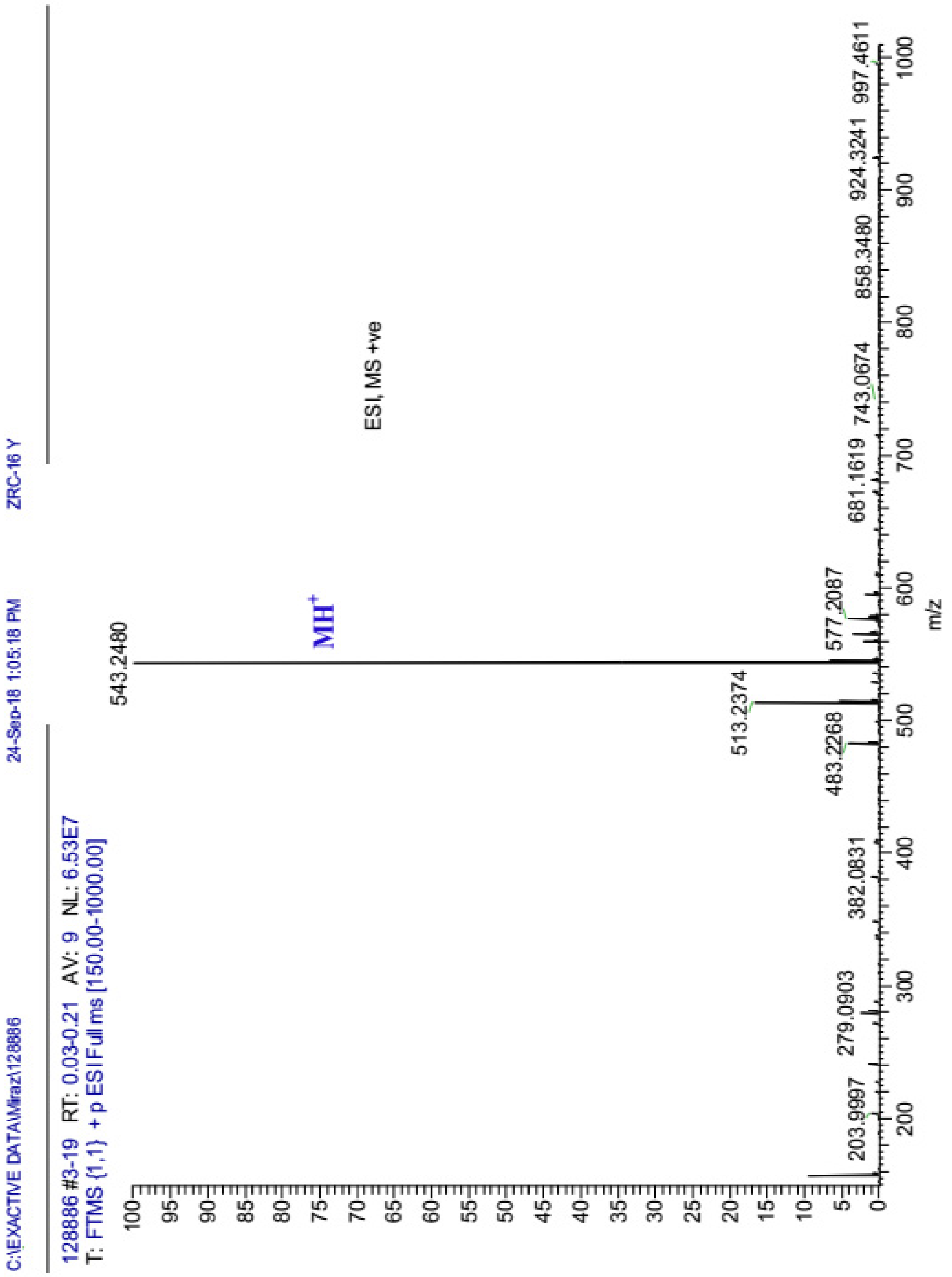

Figure S8. Mass spectrum of compound **1** (ZRC-16Y)

Characterization of Compound 2 and 3 (ZRP-14) as *N*-methylatanine and 3-dimethylallyl-4,8-dimethoxy-1-methyl-2-quinolone

ZRP-14 was isolated as a greenish gum, showed a blue fluorescent spot under UV light at 366 nm on a TLC plate and did not give any color after spraying with vanillin-sulfuric acid reagent followed by heating for 2 minutes.

The ^{1}H NMR spectrum (400 MHz, $CDCl_3$; Figure S9) of ZRP-14 indicated a mixture of two prenylated 2-quinolones, compounds 2 and 3 which were confirmed by ^{13}C NMR (Figure S10), COSY (Figure S11), HSQC (Figure S12) and HMBC (Figure S13) experiments. The ^{1}H NMR spectrum (Table S1 & Table S2) displayed two olefinic protons at δ 5.20 (2H, t, *J* = 7.0 Hz), two methylene groups at δ 3.34 and 3.33 (2H, d, *J* = 7.0, each), four methyls at δ 1.62 (6H s) and δ 1.75 (6H s), indicating the presence of two prenyl (dimethylallyl) groups in the compounds. The spectrum further revealed four adjacent aromatic protons at δ 7.76 dd (*J* = 8.0, 1.1 Hz), 7.20 ddd (*J* = 8.6, 8.0, 1.4 Hz), 7.48 ddd (*J* = (8.6, 8.4, 1.1 Hz) and 7.30 d (*J* = 8.4 Hz) comprising an ABCD ring system and three adjacent aromatic protons at δ 7.38 dd (*J* = 8.0, 1.2 Hz), 7.09 dd (*J* = 8.0, 7.9 Hz) and 6.97dd (*J* = 7.9, 1.2 Hz) comprising an ABC ring system, suggesting a di-and tri-substituted benzene rings. In addition, the ^{1}H NMR spectrum showed two N-methyl signals at δ 3.66 and 3.89 and three methoxy groups at δ 3.81, 3.82 and 3.85. The ^{13}C NMR spectrum showed 35 carbons including two carbonyl carbons at δ 164.0 and 165.0 and three oxygenated unsaturated carbons at δ 160.2, 160.0 and 148.8. Position of the prenyl groups in the two compounds was confirmed at C-3 by an HMBC experiment as methylene at 1' showed ^{2}J correlation to C-3 and ^{3}J correlation to C-2 and C-4 (Table S1 & Table S2). Further, the N-methyl groups in both of the compounds showed ^{3}J correlations to both C-2 (C=O) and C-9. All of the above data strongly suggested that ZRP-14 is a mixture of two prenylated N-methyl 2-quinolones.The methoxy group at δ 3.85 showed ^{3}J correlation to C-4 (160.2) of the di-substituted benzene ring while the remaining two methoxy groups at δ 3.81 and 3.82 showed ^{3}J correlations to C-4 (δ 160.0) and C-8 (δ 148.8) of the tri-substituted benzene ring. On the basis of above spectral data compound **2** and compound **3** were identified as 3-dimethylallyl-4-methoxy-1-methyl-2-quinolone or N-methylatanine and 3-dimethylallyl-4,8-dimethoxy-1-methyl-*2*-quinolone respectively. These two compounds (compounds **2** & **3)** were isolated for the first time from the genus *Zanthoxylum.*

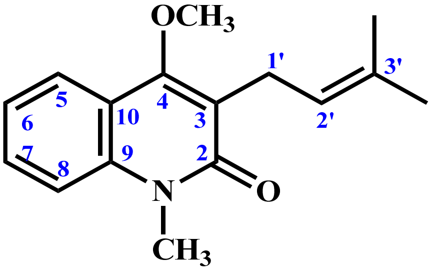

***N*-methylatanine**

Table S1. NMR spectral data ($CDCl_3$) for compound 2 (ZRP-14) [17].

Position	$δ_H$[a]	$δ_C$[b]	HSQC	HMBC
2	---	164.0		
3	---	122.6		
4	---	160.2		
5	7.76 dd (*J* = 8.0, 1.1 Hz)	123.4	123.4	130.1 (C-7), 139.0 (C-9)
6	7.20 ddd (*J* = 8.6, 8.0, 1.4 Hz)	121.9	121.9	122.4 (C-10)
7	7.48 ddd (*J* = (8.6, 8.4, 1.1 Hz)	130.1	130.1	
8	7.30 d (*J* = 8.4 Hz)	114.1	114.1	122.4 (C-10)
9	---	139.0		
10	---	122.4		
1’	3.34 d (2H, *J* = 7.0 Hz)	24.3	24.3	122.6 (C-3), 132.5 (C-3’), 160.0 (C-4), 165.0 (C-2)
2’	5.20 t (*J* = 7.0 Hz)	121.5	121.5	
3’	---	132.5		
Me-3’*cis*	1.62, 3H s	25.7	25.7	132.5 (C-3’), 122.6 (C-3), 19.0 (Me-3’*trans*)
Me-3’*trans*	1.75, 3H s	19.0	19.0	132.5 (C-3’), 122.6 (C-3), 25.7(Me-3’*cis*)
N-Me	3.66, 3H s	29.8	29.8	139.0 (C-9), 164.0 (C-2)
OMe-4	3.85, 3H s	61.8	61.8	160.2 (C-4)

[a] = measured in 400 MHz, b= measured in 100 MHz

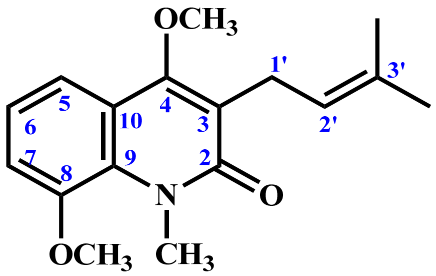

3-dimethylallyl-4,8-dimethoxy-1-methyl-*2-quinolone*

Table S2. NMR spectral data ($CDCl_3$) for compound 3 (ZRP-14) [17].

Position	δ_H[a]	δ_C[b]	HSQC	HMBC
2	---	165.0		
3	---	122.6		
4	---	160.0		
5	7.38 dd(*J* = 8.0,1.2 Hz)	116.0	116.0	130.6 (C-9), 113.5 (C-7)
6	7.09 dd (*J* = 8.0, 7.9 Hz)	122.7	122.7	120.2 (C-10), 148.8 (C-8)
7	6.97 dd (*J* = 7.9, 1.2 Hz)	113.5	113.5	130.6 (C-9), 116.0 (C-5)
8	---	148.8		
9	---	130.4		
10	---	120.2		
1’	3.33 d (2H, *J* = 7.0 Hz)	24.4	24.4	122.6 (C-3), 132.5(C-3’), 160.0 (C-4), 165.0 (C-2)
2’	5.20 t (*J* = 7.0 Hz)	121.5	121.5	
3’	---	132.5		
Me -3’ *cis*	1.62, 3H s	25.7	25.7	132.5 (C-3’), 122.6(C-3), 19.0 (Me-3’*trans*)
Me-3’*trans*	1.75, 3H s	19.0	19.0	132.5 (C-3’) , 122.6 (C-3), 25.7(Me-3’*cis*)
N-Me	3.89, 3H s	35.5	35.5	130.4 (C-7), 165.0 (C-2)
OCH_3-4	3.81, 3H s	61.6	61.6	160.0 (C-4)
OCH_3-8	3.82, 3H s	56.7	56.7	148.8 (C-8)

[a] = measured in 400 MHz, b= measured in 100 MHz

NMR spectrum of compound 2&3:

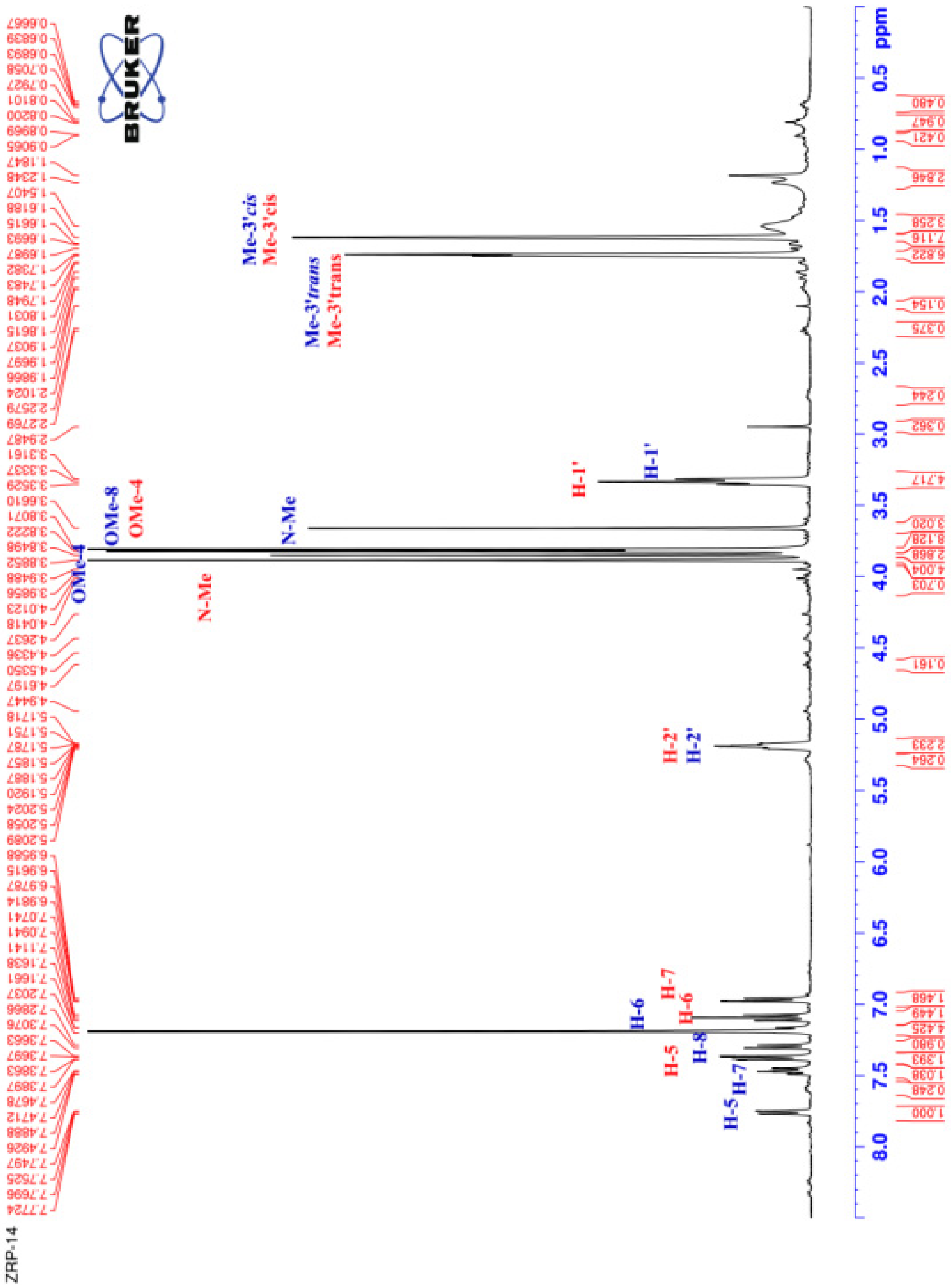

Figure S9. ^{1}H NMR (400 MHz, $CDCl_3$) spectrum of compound **2** and **3** (ZRP-14)

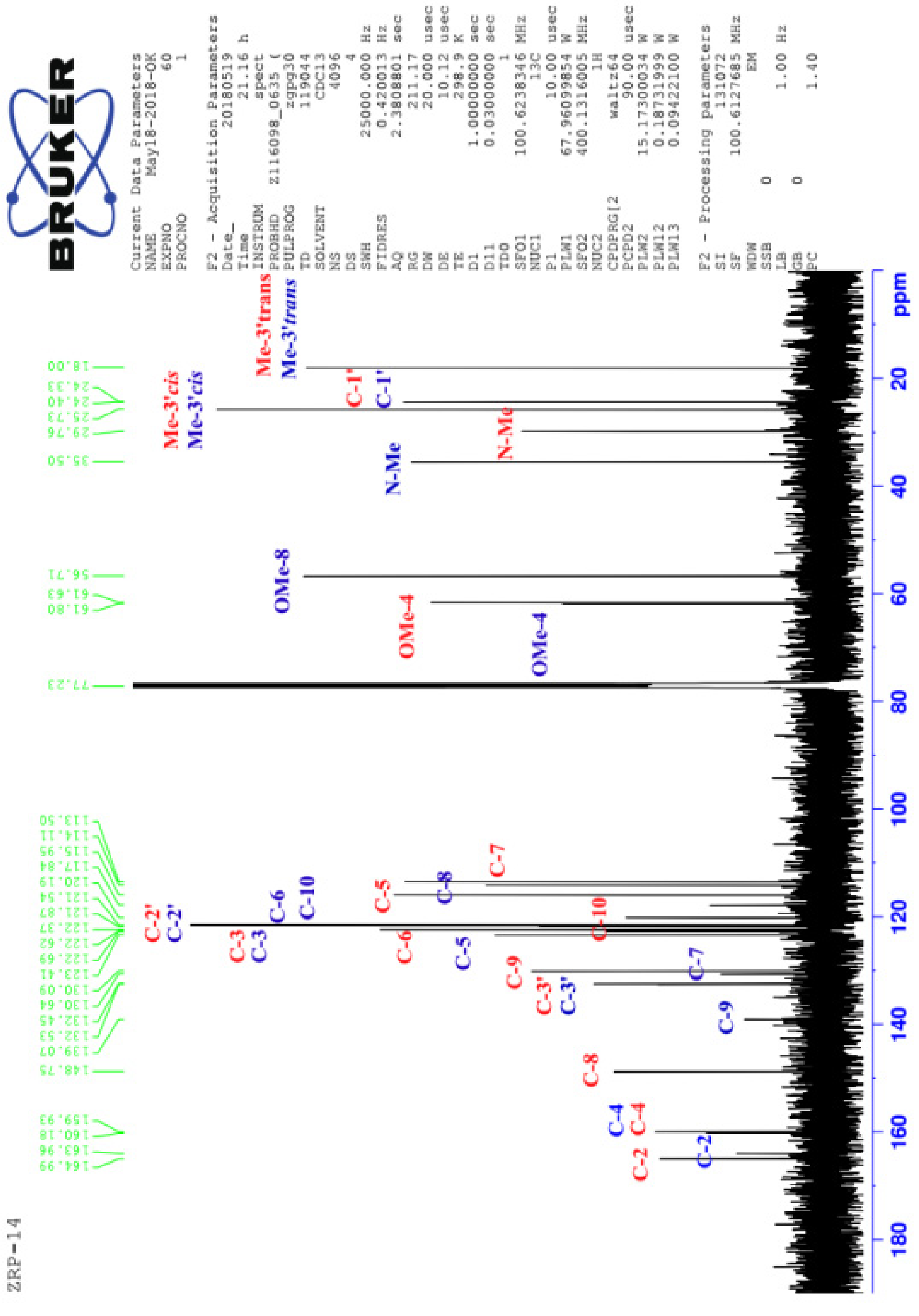

Figure S10. ^{13}C NMR (100 MHz, $CDCl_3$) spectrum of compound **2** and **3** (ZRP-14)

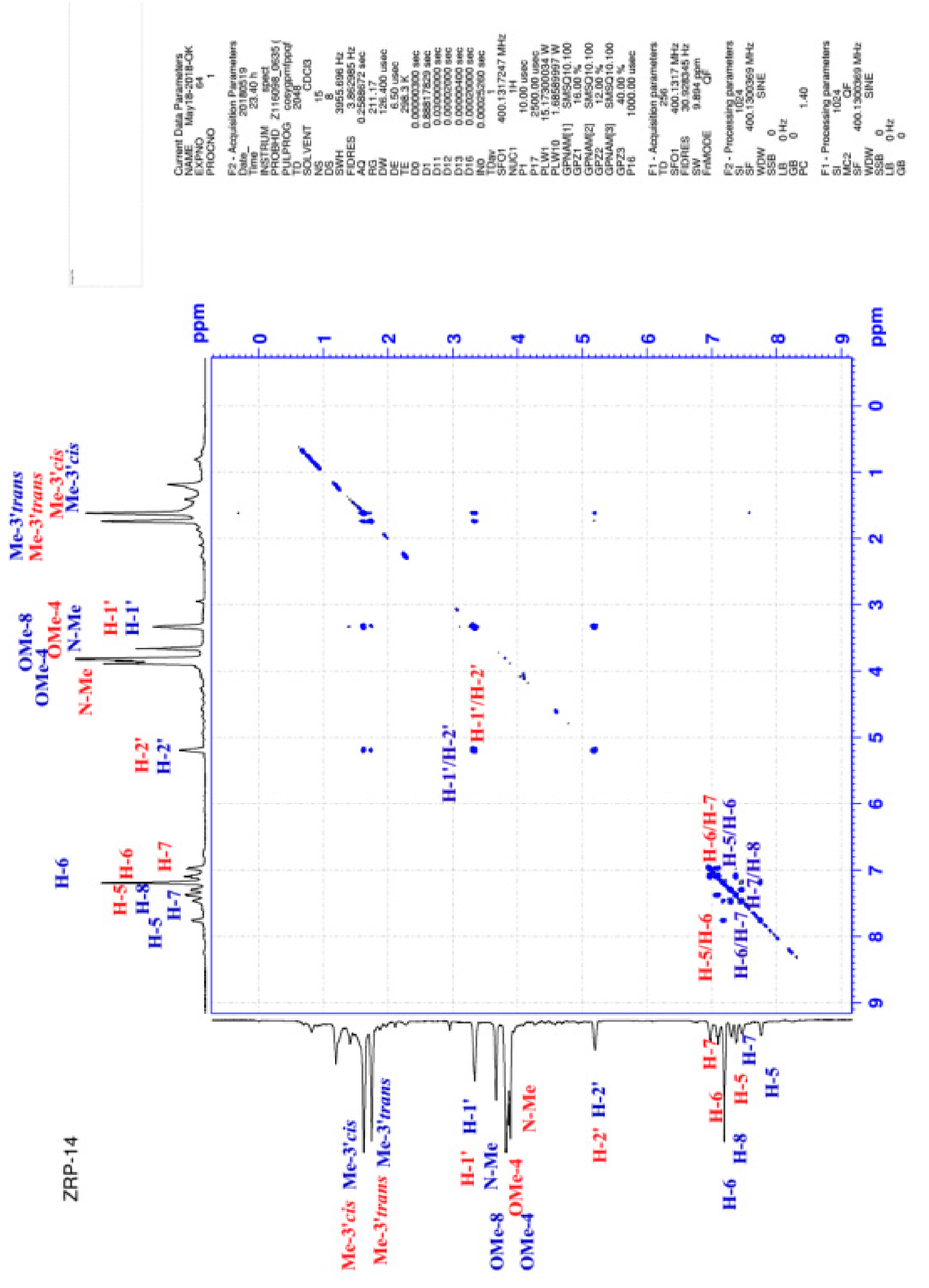

Figure S11. COSY (400 MHz, $CDCl_3$) spectrum of compound **2** and **3** (ZRP-14)

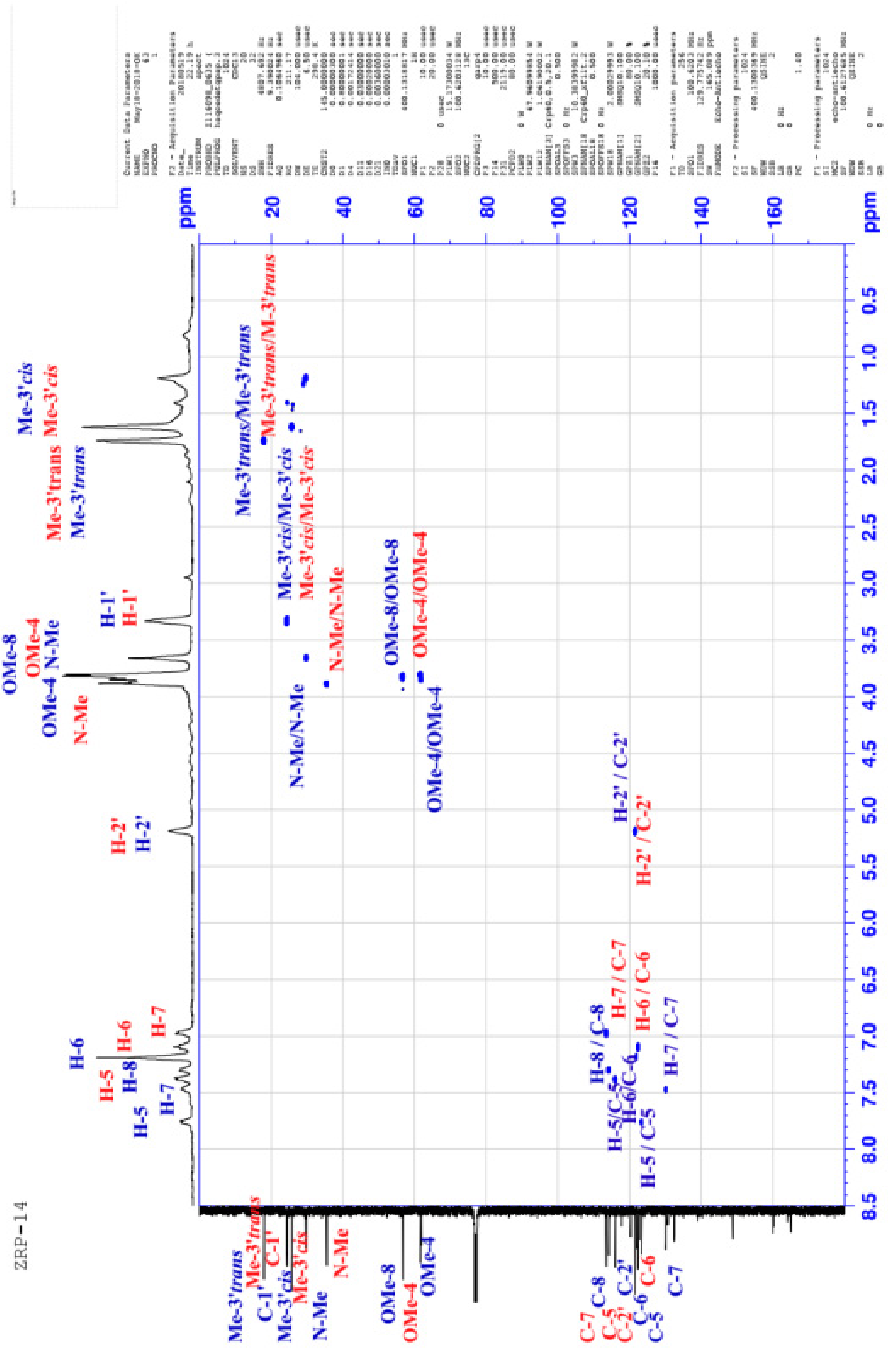

Figure S12. HSQC (400 MHz, $CDCl_3$) spectrum of compound **2** and **3** (ZRP-14)

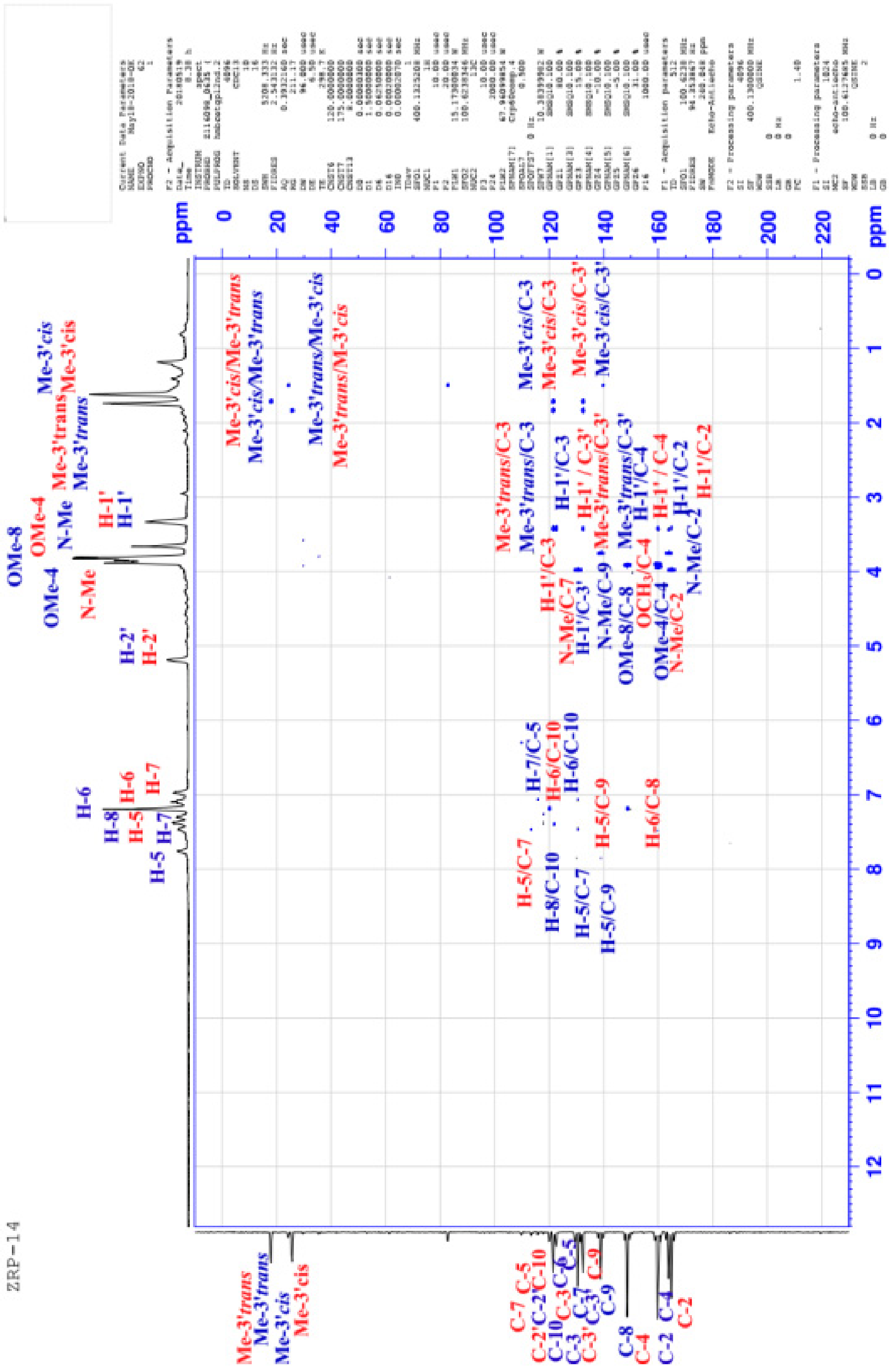

Figure S13. HMBC (400 MHz, CDCl3) spectrum of compound **2** and **3** (ZRP-14)

Characterization of compound 4 (ZRC-7) as 8-O-demethylchelerythrine

Compound **4**, isolated as yellow mass, showed dark quenching spot under UV light at 254 nm on a TLC plate and gave yellow color when sprayed with vanillin-sulfuric acid reagent followed by heating at 110 ºC for 2 minutes.

The ^{1}H NMR spectrum (Table S3, Figure S14) of this compound displayed two sets of ortho-coupling doublets at δ 7.99 (J = 9.0 Hz), 8.67 (J = 9.0 Hz), 8.67 (J = 9.0 Hz) and 8.24 (J = 9.0 Hz) and two singlets at δ 7.58 and 8.31 attributable to H-9, H-10, H-11, H-12, H-1 and H-4 respectively. In addition, the spectrum showed one methoxy group at δ 4.28, an N-methyl at δ 5.50 and a methylenedioxy group at δ 6.29. A highly deshielded proton at δ 9.71 could be assigned to H-6, the deshielded effect may be due to presence of neighbouring nitrogen atom. On the basis of above data compound **4** was identified as 8-O-demethylchelerythrine. The ^{1}H NMR data were formed similar to those reported in the literature [18]. 8-O-demethylchelerythrine is reported for the first time from *Z. rhetsa*.

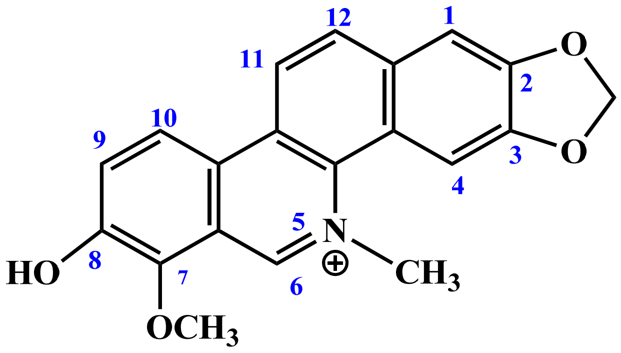

8-O-demethylchelerythrine

Table S3. ^{1}H NMR spectral data (400 MHz, CD_3OD) for compound 9 (ZRC-7)

	**Compound 9**	**8-O-demethylchelerythrine*[18]**
**Position**	**δ_H**	**δ_H**
1	7.58 s	7.50 s
4	8.31 s	7.94 s
6	9.71 s	9.69 s
9	7.99 d (J = 9.0 Hz)	8.05 d (J = 9.1 Hz)
10	8.67 d (J = 9.0 Hz)	8.50 d (J = 9.1 Hz)
11	8.67 d (J = 9.0 Hz)	8.50 d (J = 9.1 Hz)
12	8.24 d (J = 9.0 Hz)	8.19 d (J = 9.1 Hz)
OMe-7	4.28 3H s	4.25 3H, s
N-Me	5.51 3H s	5.00 3H, s
$-OCH_2O-$	6.29 2H s	6.21 2H, s

* = spectrum recorded in $CDCl_3$-TFA

NMR spectrum of compound 4 characterized as 8-O-demethylchelerythrine:

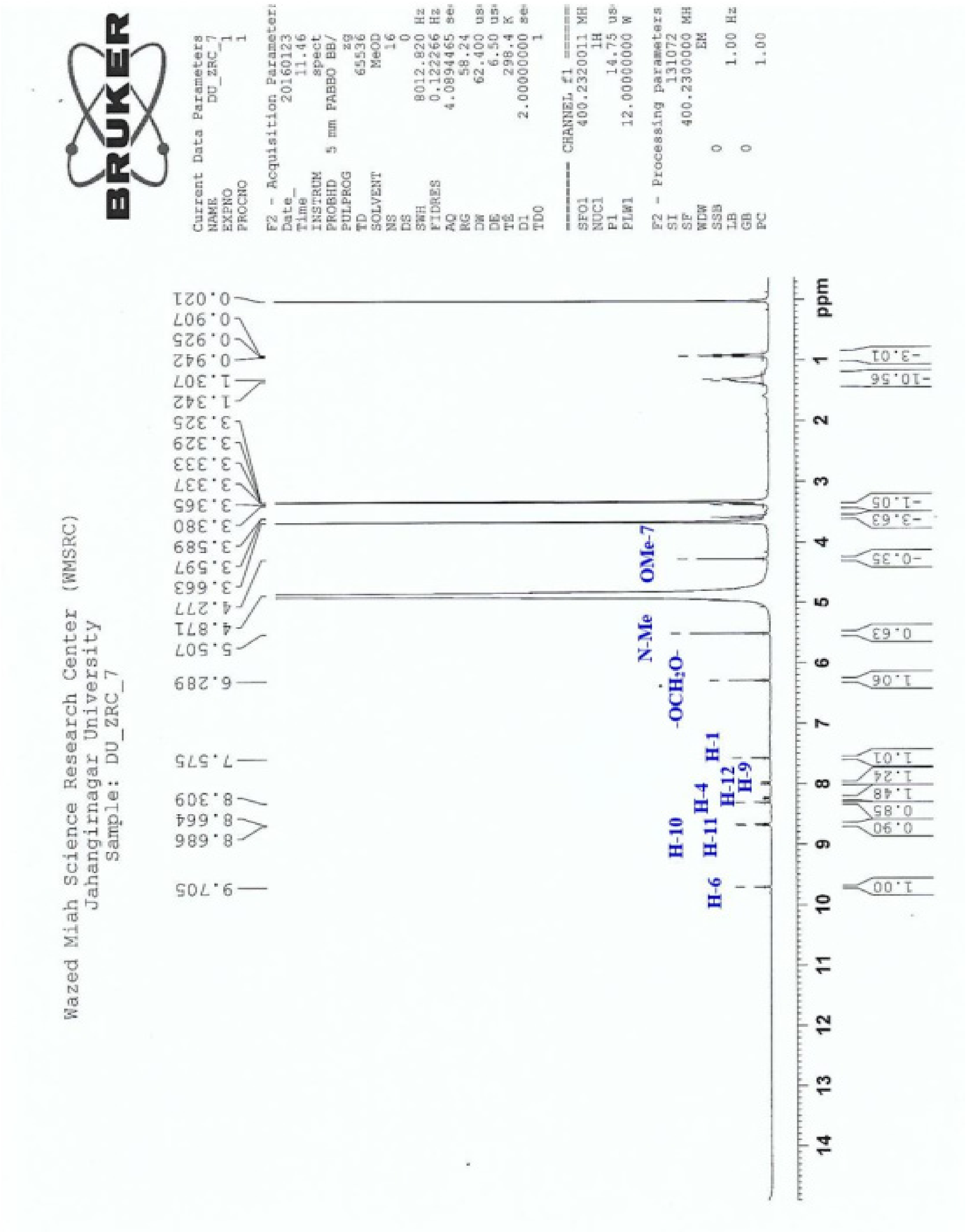

Figure S14 ^{1}H NMR (400 MHz, $CDCl_3$) spectrum of compound **4** (ZRC-7)

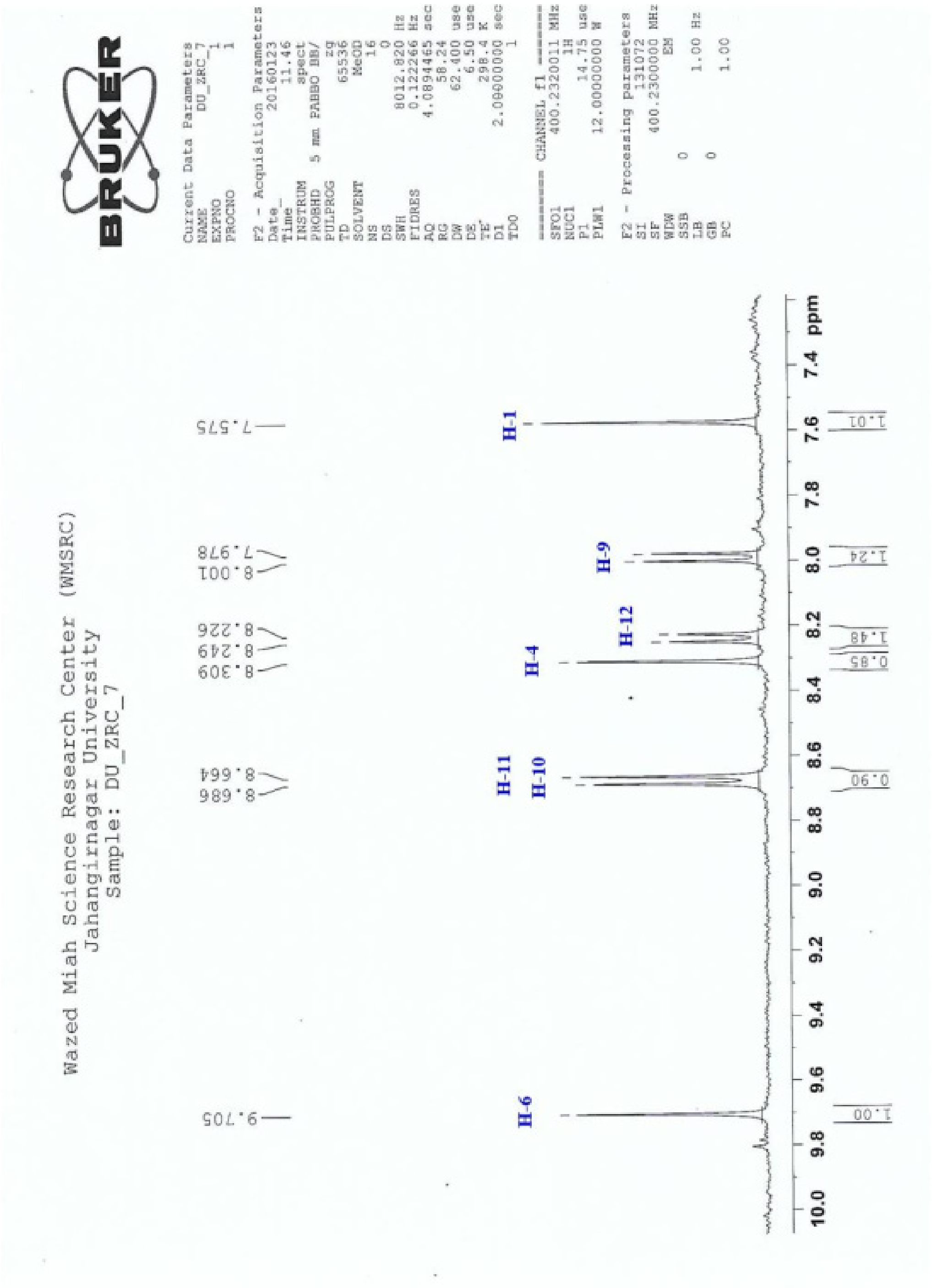

Figure S15 partially expanded ^{1}H NMR (400 MHz, $CDCl_3$) spectrum of compound **4** (ZRC-7)

Characterization of compound 5 and 6 (ZRC-79) as mixture of chelerythrine and 7-methoxynitidine

ZRC-79, isolated as yellow mass, presented a deep yellow fluorescent spot at 366 nm UV light on a TLC plate and produced reddish brown color with Dragendorff's reagent.

The ^{1}H NMR spectrum (400 MHz, $CDCl_3$; Table S4; Figure S16) of ZRC-79 indicated the presence of two compounds in 2:1 ratio. The major signals include two pairs of ortho coupling doublets at δ 7.85, 8.34, 8.32 and 7.98, two aromatic proton singlets at δ 7.32 & 8.02, two methoxy groups at δ 4.41 and 4.04, a methylenedioxy group at δ 6.18 (2H, s), a very deshielded singlet at δ 10.72 and an N-methyl group resonating at δ 5.27 (3H, s). All these ^{1}H NMR signals were found identical to those reported for chelerythrine [19].

The rest of the ^{1}H NMR signals (minor compound) (Table S5) include two ortho-coupling doublets at δ 8.23 and 7.85, three aromatic proton singlets at δ 7.82, 8.04 and 8.42, three methoxy groups at δ 4.41, 4.04 and 3.88, a methylenedioxy group at δ 6.22 (2H, s), a deshielded proton singlet at δ 10.72 and an N-methyl at δ 5.24 (3H, s). All these latter signals indicated the minor compound similar to chelerythrine (major one) but with additional methoxy group instead of the proton at C-9. Thus ZRC-79 was identified as a mixture of chelerythrine (compound **5)** and 7-methoxynitidine, (compound **6**). 7-methoxynitidine is a previously undescribed naturally occuring benzophenanthredine alkaloid, however, it was synthesized by Ishii et al in 1985 [20].

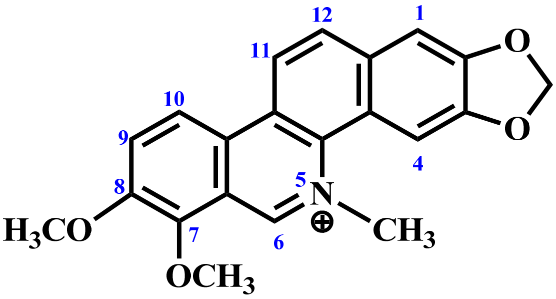

Chelerythrine

Table S4. ^{1}H NMR spectral data (400 MHz, $CDCl_3$) for compound 5 (ZRC-79)

	**Compound 5**	**Chelerythrine [19]**
**Position**	**δ_H**	**δ_H**
1	7.32 s	7.49 s
4	8.02 s	8.08 s
6	10.72 s	9.92 s
9	7.85 d (*J* = 9.0 Hz)	8.01 d (*J* = 9.0 Hz)
10	8.34 d (*J* = 9.0 Hz)	8.60 d (*J* = 9.0 Hz)
11	8.32 d (*J* = 9.0 Hz)	8.56 d (*J* = 9.0 Hz)
12	7.98 d (*J* = 9.0 Hz)	8.10 d (*J* = 9.0 Hz)
OMe-7	4.41 3H s	4.27 3H s
OMe-8	4.04 3H s	4.12 3H s
N-Me	5.27 3H s	4.97 3H s
-OCH_2O-	6.18 2H s	6.26 2H s

*= spectrum recorded in CD_3OD

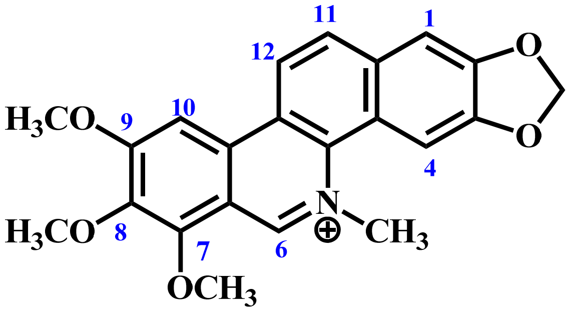

7-methoxynitidine

Table S5. ^{1}H NMR spectral data (400 MHz, $CDCl_3$) for compound 6 (ZRC-79)

Position	δ_H
1	7.82 s
4	8.04 s
6	10.72 s
10	8.42 s
11	8.23 d (J = 9.0 Hz)
12	7.85 d (J = 9.0 Hz)
OMe-7	4.41 3H s
OMe-8	4.04 3H s
OMe-9	3.88 3H s
N-Me	5.24 3H s
-OCH_2O-	6.22 2H s

NMR spectrum of compound 5 and 6 characterized as a mixture of chelerythrine and 7-methoxynitidine:

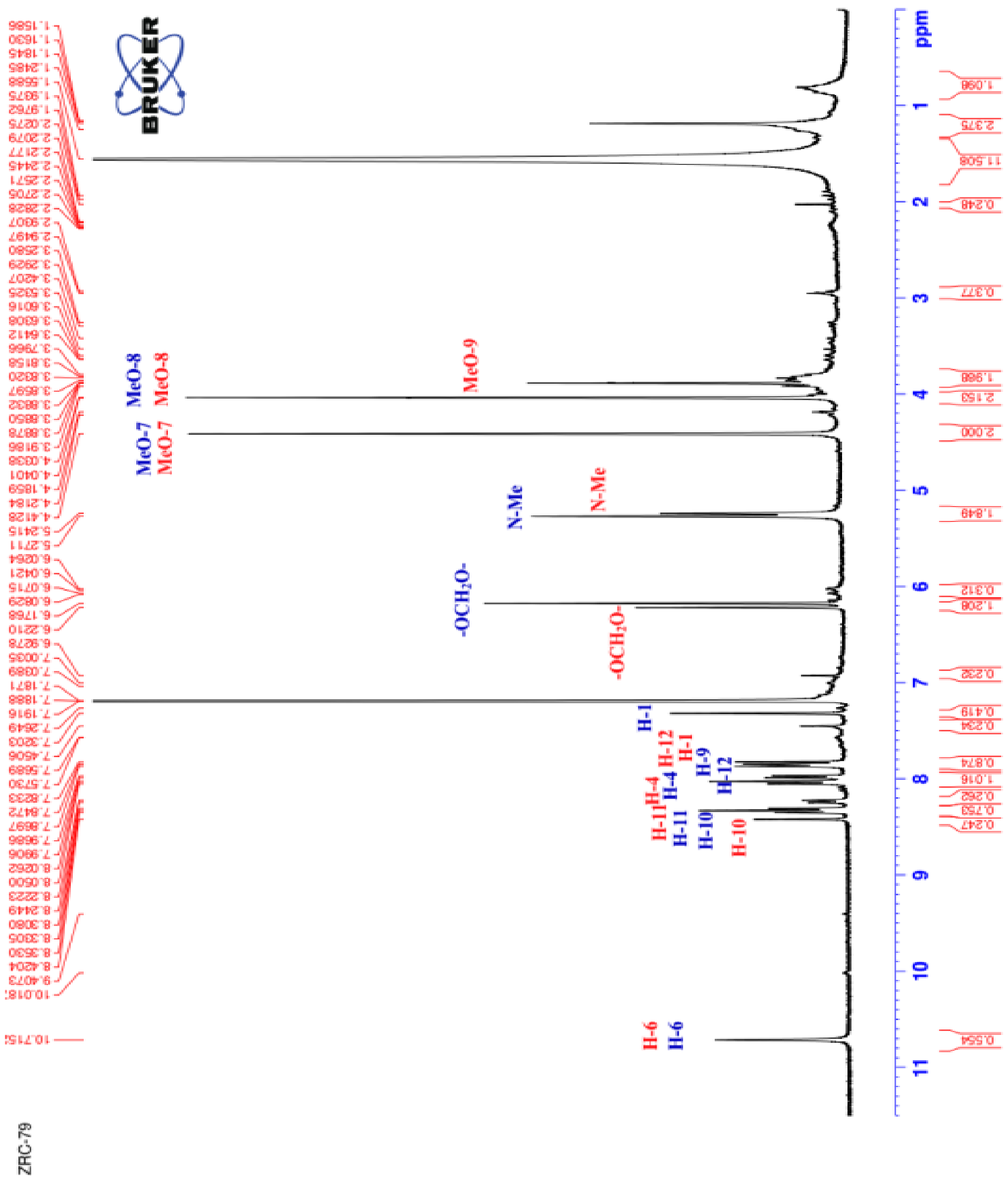

Figure S16. ^{1}H NMR (400 MHz, $CDCl_3$) spectrum of compound **5** & **6** (ZRC-79)

Characterization of compound 7 (ZRC-35) as canthin-6-one

Compound **7**, isolated as colorless crystals, provided blue fluorescent spot on a TLC plate at 366 nm UV light. The ^{1}H NMR spectral (400 MHz, $CDCl_3$; Table S6, Figure S17) data of compound **7** demonstrated the presence of four aromatic protons at δ 8.17 (d, *J* = 8.0 Hz), 7.59 (dd, *J* = 8.0, 7.5 Hz), 7.81 (dd, *J* = 8.4, 7.5 Hz) and 8.65 (d, *J* = 8.4 Hz) suggesting a disubstituted benzene ring and could be assigned to H-8, H-9, H-10 and H-11 respectively. Two ortho coupled aromatic protons resonating at δ 8.20 (d, *J* = 5.9 Hz) and δ 8.73 (d, *J* = 5.9 Hz) were attributable to H-1 and H-2 respectively. Another set of doublets at δ 8.49 (*J* = 10 Hz) and δ 7.09 (*J* = 10 Hz) were attributable to H-4 and H-5 of the conjugated lactam ring of canthin-6-one. The structure of compound **7** was identified as canthin-6-one by comparing its spectroscopic data with those published for the compound [21].

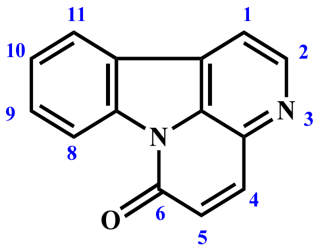

Canthin-6-one

Table S6 NMR spectral data (400 MHz; $CDCl_3$) for compound 7 (ZRC-35)

Position	Compound 7<br>δ_H	Canthin-6-one [21]<br>δ_H
1	8.20 d (*J* = 5.9 Hz)	8.35 d (*J* = 4.8 Hz)
2	8.73 d (*J* = 5.9 Hz)	8.86 d (*J* = 4.8 Hz)
4	8.49 d (*J* = 10 Hz)	8.7 d (*J* = 9.7 Hz)
5	7.09 d (*J* = 10 Hz)	7.02 d (*J* = 9.7 Hz)
8	8.17 d (*J* = 8.0 Hz)	8.55 d (*J* = 8.1 Hz)
9	7.59 dd (*J* = 8.0, 7.5 Hz)	7.79 t (*J* = 7.6 Hz)
10	7.81 dd (*J* = 8.4, 7.5 Hz)	7.62 t (*J* = 7.6 Hz)
11	8.65 d (*J* = 8.4 Hz)	8.42 d (*J* = 7.8 Hz)

NMR spectrum of compound 7 characterized as canthin-6-one:

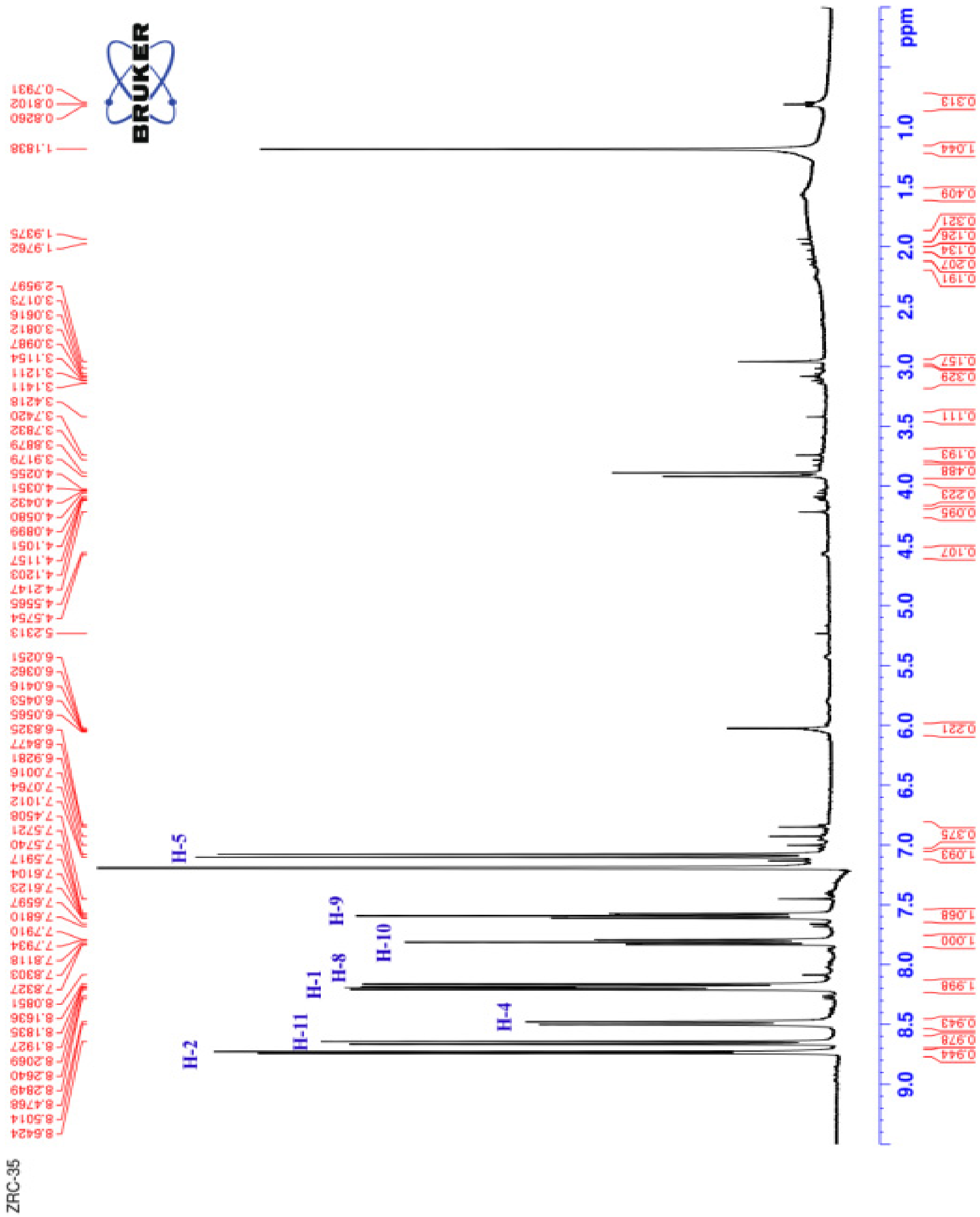

Figure S17 ^{1}H NMR (400 MHz, $CDCl_3$) spectrum of compound **7** (ZRC-35)

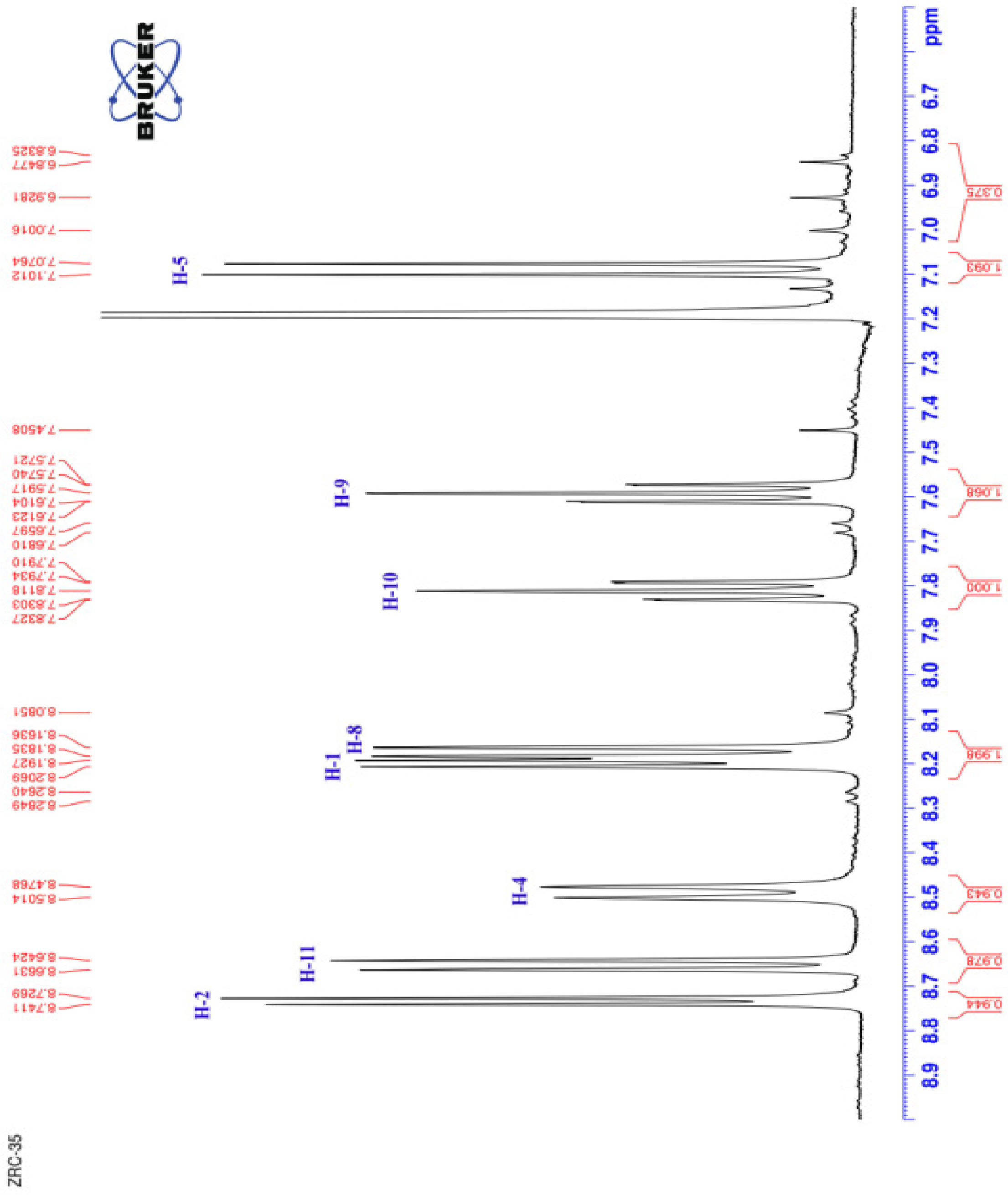

Figure S18 Partially expanded ^{1}H NMR (400 MHz, $CDCl_3$) spectrum of compound **7** (ZRC-35**)**

Characterization of compound 8 [ZRP-14(2)] as (+)-piperitol-γ-γ- dimethylallylether

Compound **15**, isolated as a yellow mass, showed blue fluorescence at 366 nm UV light on a TLC plate and produced no colour after spraying with vanillin-sulfuric acid reagent.

The ^{1}H NMR spectrum (400 MHz, $CDCl_3$; Table S7, Figure S19) of compound **15** displayed six aromatic protons at δ 6.97, 6.71, 6.76, 6.83, 6.74 and 6.76, the coupling constant of which indicated two trisustituted benzene rings. A two proton singlet at δ 5.88 and three proton singlet at δ 3.80 indicating respectively a methylenedioxy and a methoxy groups. An oxymethylene group at δ 4.50 (2H, d, J = 9.6 Hz), an olefinic proton δ 5.44 (1H, brt, J = 6.0 Hz) and two methyls at δ 1.69 and 1.65 indicated the presence of a 3-methyl-but-2-enyloxy (prenyloxy) chain in the molecule. In addition the spectrum showed eight olefinic protons at δ 3.09 to 4.44, suggesting a lignan with a furofuran ring. The ^{13}C NMR spectrum (100 MHz, $CDCl_3$; Table S7, Figure S20) showed twenty-four carbons including a methylenedioxy carbon at δ 101.1, a methoxy carbon at δ 56.0 and five carbinol carbon at δ 85.9, 85.8, 71.7, 71.7 and 65.8. A methylenedioxy group at δ 5.88 with three aromatic protons suggested the presence of a 3, 4-methylenedioxyphenyl (i.e piperonyl) group. The rest three aromatic protons, together with the 3-methyl-but-2-enyloxy (prenyloxy) and the methoxy group indicated the presence of a 3-methoxy-4-prenyloxyphenyl group. The HSQC (Figure S21) and HMBC (Figure S22) experiment allowed assignment of all the protons and carbons in the molecule. All these data enabled the identity of ZRP-14(2) as a furofuran lignan with a piperonyl group and a 3-methoxy-4-prenyloxy group. On the basis of above spectral data, compound **8** was identified as (+)-piperitol-γ-γ-dimethylallylether.

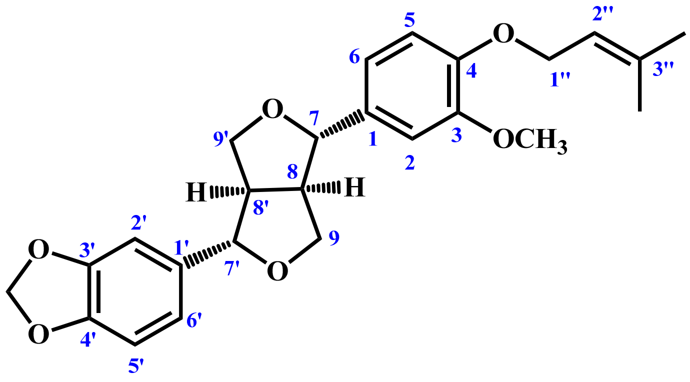

(+)- Piperitol-γ-γ-dimethylallylether

Table S7. NMR spectral data ($CDCl_3$) for compound 8 [ZRP-14(2)]

Position	δ_H[a]	δ_C[b]	HSQC	HMBC
1		135.1		
2	6.97 d (*J* = 1.2 Hz)	106.5	108.4	148.0 (C-4)
3		147.9		
4		148.0		
5	6.71 d (*J* = 7.0, 9.0 Hz)	112.9	114.2	135.1 (C-1)
6	6.76 dd (*J* = 8.0,2.0 Hz)	119.4	118.4	
7	4.66 d (*J* = 5.2 Hz)	85.8	82.1	71.7 (C-9), 71.7 (C-9’)
8	3.09 m	54.4	50.2	
9α	4.25 dd (*J* = 9.6, 6.0 Hz)	71.7	69.8	85.8 (C-7), 85.9 (C-7’)
9β	3.94 m			85.8 (C-7), 85.9 (C-7’)
1’		133.5		
2’	6.83br s	109.4	106.5	149.8 (C-4’)
3’		147.1		
4’		149.8		
5’	6.74 d (*J* = 8.8 Hz)	108.2	108.2	133.5 (C-1’)
6’	6.76 d (*J* = 8.0 Hz)	118.2	119.5	
7’	4.44 d (*J* = 4 Hz)	85.9	87.7	71.7 (C-9), 71.7 (C-9’)
8’	3.09 m	54.1	54.6	
9’α	4.25 dd (*J* =9.6, 6.0 Hz)	71.7		85.8 (C-7), 85.9 (C-7’)
9’β	3.94 m			85.8 (C-7), 85.9 (C-7’)
OMe-3	3.80 3H s	56.0	56.0	147.9 (C-3)
3’,4’-OCH_2O-	5.88 2H s	101.1	101.0	147.1 (C-3’), 149.8 (C-4’)
1’’	4.50 (*J* =9.6 Hz)	65.8		120.0 (C-2’’), 137.6 (C-3’’)
2’’	5.44 (*J* =6 Hz)	120.0		
3’’		137.6		
Me-3’’*cis*	1.69 3H s	25.8		18.2 (Me-3’’*trans*), 120.0 (C-2’’), 137.6 (C-3’’)
Me-3’’*trans*	1.65 3H s	18.2		25.8(Me-3’’*cis*), 120.0 (C-2’’), 137.6 (C-3’’)

[a] = measured in 400 MHz, b= measured in 100 MHz

NMR spectrum of compound 8 as (+)-piperitol-γ-γ-dimethylallylether:

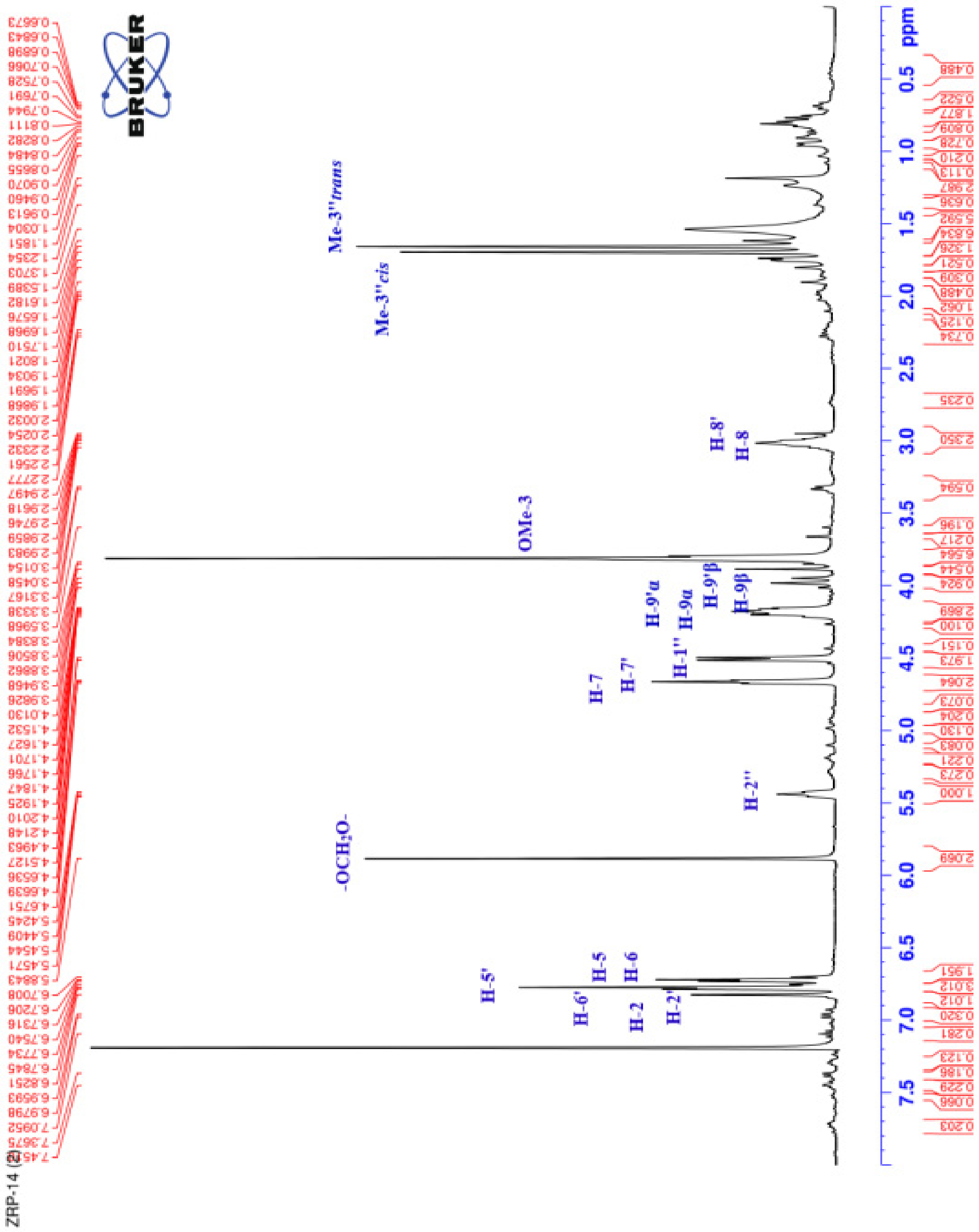

Figure S19. ^{1}H NMR (400 MHz, $CDCl_3$) spectrum of compound **8** [ZRC-14 (2)]

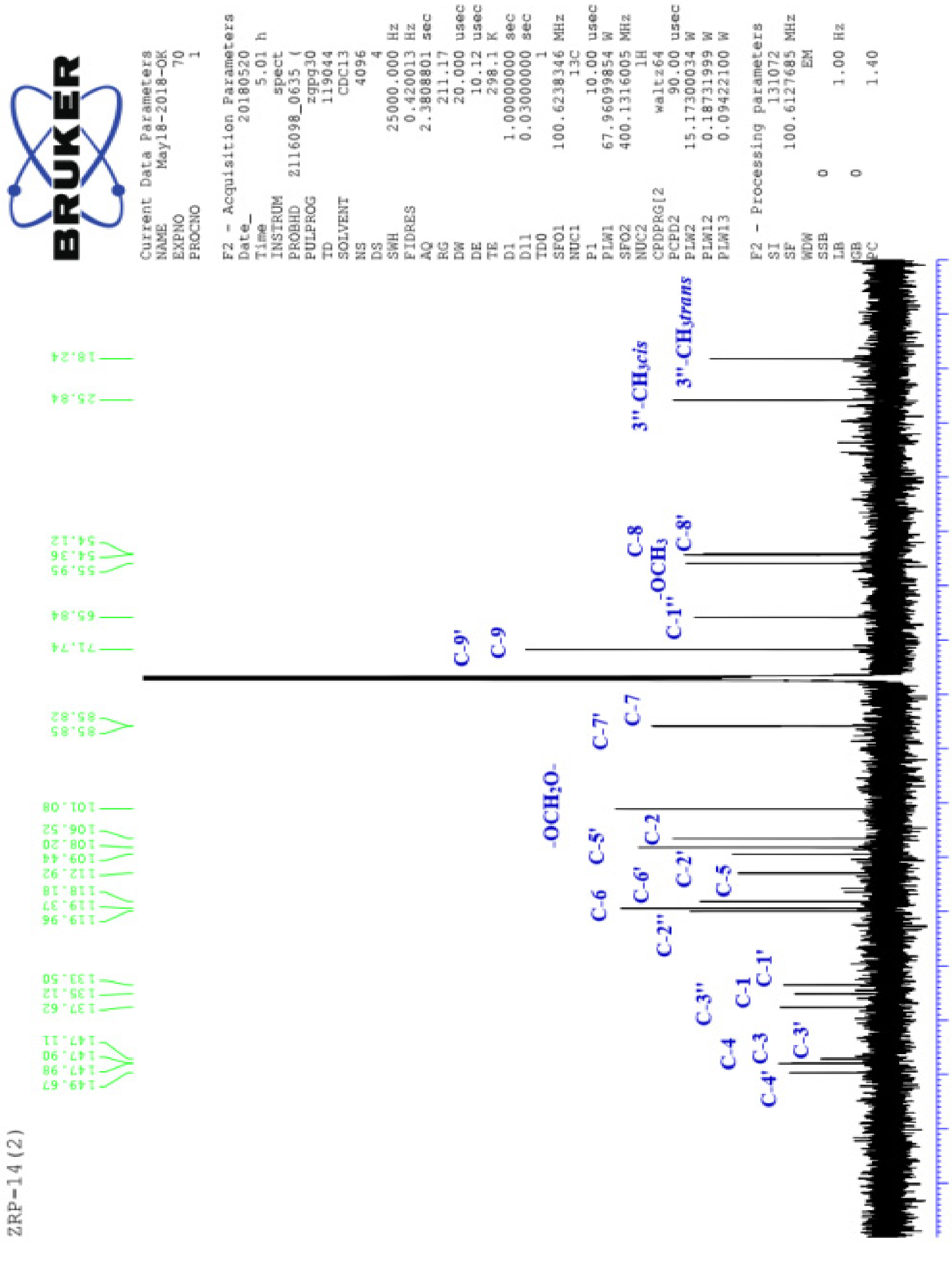

Figure S20. ^{13}C NMR (100 MHz, $CDCl_3$) spectrum of compound **8** [ZRC-14 (2)]

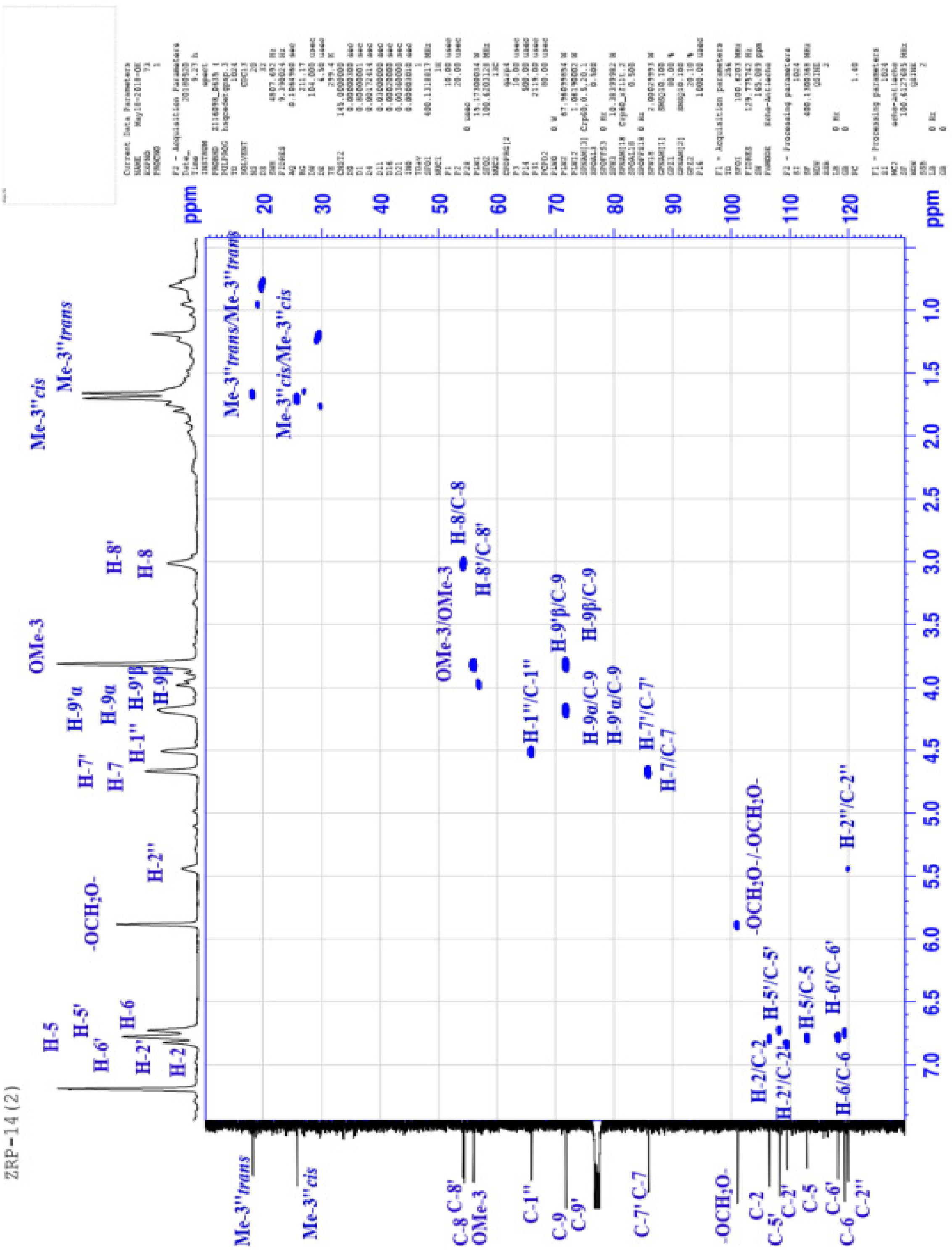

Figure S21. HSQC (400 MHz, $CDCl_3$) spectrum of compound **8** [ZRC-14 (2)]

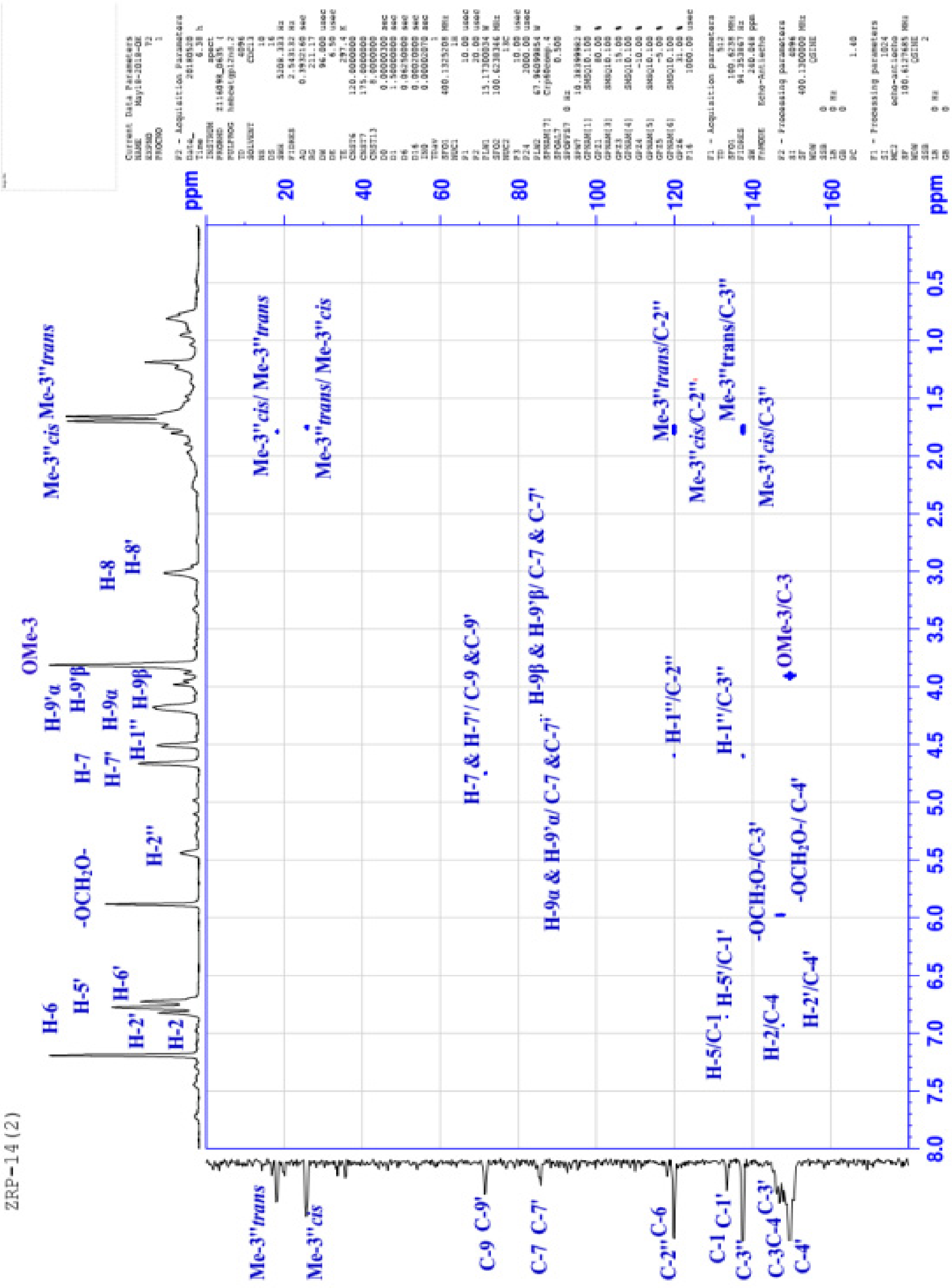

Figure S22. HMBC (400 MHz, $CDCl_3$) spectrum of compound **8** [ZRC-14 (2)]

Characterization of compound 9 and compound 10 (ZRP-51) as mixture of β-sitosterol and stigmasterol

ZRP-51 was isolated as colorless crystals and produced purple color when sprayed with vanillin in sulphuric acid reagent, followed by heating for 2 minutes. The compounds were appeared as a single spot on a TLC plate and therefore could not be separated from each other.

The ^{1}H NMR spectrum (Table S8; Figure S22) showed two singlets at δ 0.70 and 1.03, assignable to H-18 and H-19 and the other three doublets at δ 0.95, 0.84 and 0.86 having a coupling constant of J = 6.4, 7.2 and 7.2 Hz which could be attributed to H-21, H-26 and H-27 respectively. Similarly, in the spectrum a triplet at δ 0.87 (J = 7.2 Hz) was assigned to H-29. The spectrum also showed an olefinic proton at δ 5.37 with coupling constant J = 5.2 Hz and a multiplet at δ 3.55, assignable to H-6 and H-3 of a sterol moiety. Thus compound **10** was identified as β–sitosterol. The structure was further confirmed by comparing its ^{1}H NMR data with those published [22].

In addition to the signals discussed for β–sitosterol, the ^{1}H NMR spectrum (Table S8, Figure S22) also displayed two olefinic protons at δ 5.18 and 5.04 (dd, J= 15.2, 8.6 Hz each) respectively assigned to H-22 and H-23. Three doublets at δ 1.04, 0.83 and 0.88 with coupling constant J = 7.5, 7.0 and 6.3 Hz attributed to H-21, H-26 and H-27 respectively. The ^{1}H NMR also showed a triplet at δ 0.83 (J = 7.0 Hz) was assigned to H-29 and two singlets at δ 0.72 and 1.03, assignable to H-18 and H-19. The spectrum as well showed a doublet of an olefinic proton at δ 5.37 (d, J = 5.2 Hz) and a multiplet at δ 3.55, assignable to H-6 and H-3 of a sterol moiety. These ^{1}H NMR data were found to be in close agreement to those reported for stigmasterol [22].Thus compound **9** was identified as stigmasterol.

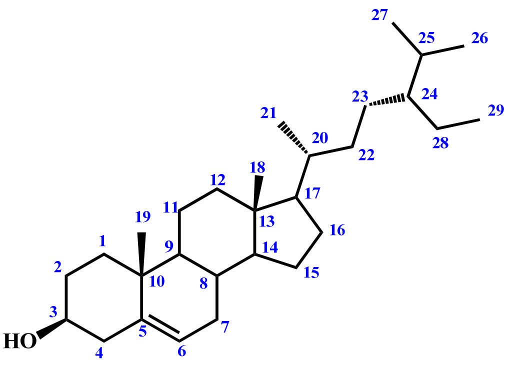

β-sitosterol

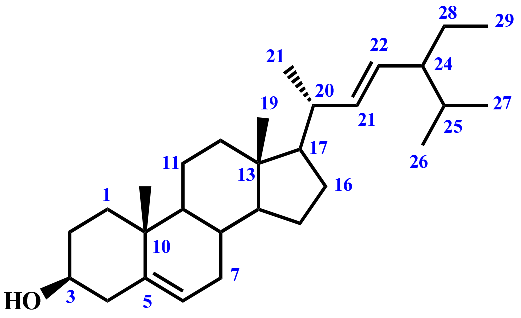

Stigmasterol

Table S8. ^1H NMR spectral data (400 MHz, $CDCl_3$) for compound 10 and 9 (ZRP-51)

Position	Compound 10	β-sitosterol [22]	Compound 9	Stigmasterol [22]
	δ_H	δ_H	δ_H	δ_H
3	3.55 m	3.53 m	3.55 m	3.52 m
6	5.37 d (*J* = 5.2 Hz)	5.37 br s	5.37 d (*J* = 5.2 Hz)	5.38 br s
18	0.70 3H, s	0.68 3H, s	0.72 3H, s	0.69 3H, s
19	1.03 3H, s	1.01 3H, s	1.03 3H, s	1.01 3H, s
21	0.95 d (*J* = 6.4 Hz)	0.92 d (*J* =6.4 Hz)	1.04 d (*J* = 7.5 Hz)	1.02 d (*J* =7.5 Hz)
22			5.18 dd (*J* = 15.2, 8.6 Hz)	4.98 1H, m
23			5.04 dd (*J* = 15.2, 8.6 Hz)	5.14 1H, m
26	0.84 d (*J* = 7.2 Hz)	0.81 d (*J* =6.4 Hz)	0.83 d (*J* = 7.0 Hz)	0.79 d (*J* =6.5 Hz)
27	0.86 d (*J* = 7.2 Hz)	0.83 d (*J* =6.4 Hz)	0.88 d (*J* = 6.3 Hz)	0.85 (d, *J* =6.5 Hz)
29	0.87 t (*J* =7.2 Hz)	0.85 t (*J* =7.5)	0.83 t (*J* = 7.0 Hz)	0.80 (t, *J* =7.5 Hz)

NMR spectrum of compound 9 and 10 as a mixture of β-sitosterol and stigmasterol:

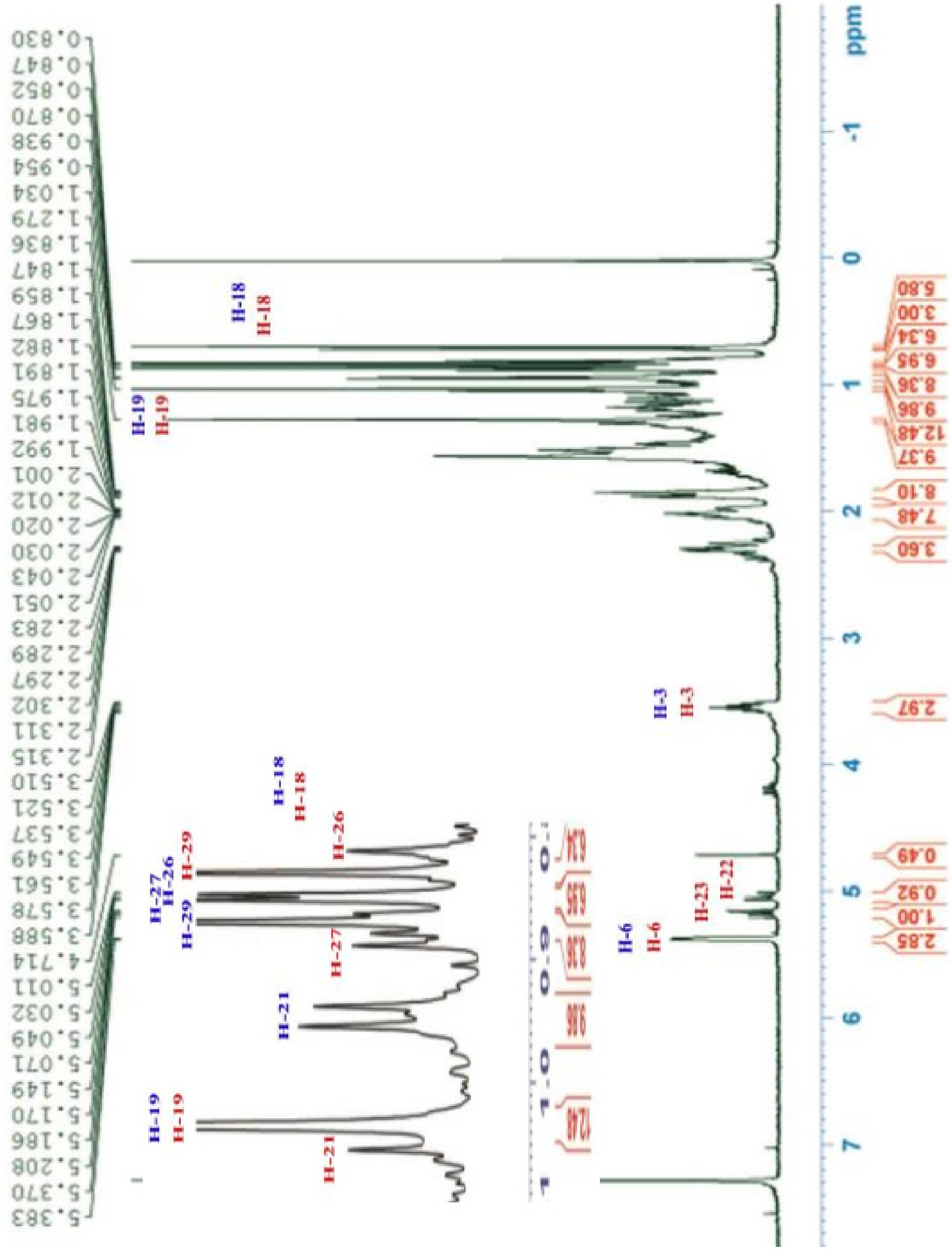

Figure S22. ^{1}H NMR (400MHz, $CDCl_3$) spectrum of compound **9** & **10** (ZRP-51)

Characterization of compound 11, compound 12 and compound 13 (ZRP-3) as a mixture of methyl oleate, methyl stearate and stearic acid

Compound **11**, **12** and **13** were obtained as light yellowish liquid, and produced grayish yellow color on a TLC plate when sprayed with vanillin in sulphuric acid reagent followed by heating for 2 minutes. The ^{1}H NMR spectral data (500 MHz, $CDCl_3$; Table S9, Figure S23) showed two olefinic proton multiplets at δ 5.36 and a methyl triplet at δ 0.88, which could be assigned to at H-9, H-10 and the terminal methyl group H-18 respectively. The protons resonating at δ 2.03 (4H, m) are the allylic protons (<u>CH_2</u>-CH=CH) H-8 and H-11. The protons directly adjacent to the carbonyl group resonated at δ 2.32 (2H, t, *J* = 7.4 Hz, H-2) and the HOOC-CH_2-<u>CH_2</u> protons resonated at δ 1.63 (2H, m, H-3). The methylene protons of the fatty chain appeared at δ 1.27 (20H, m) assignable to H-4 to H-7 and H-12 to H-17. A methoxy group at δ 3.68 indicated an esterified fatty acid. All these data permitted the identification of ZRP-3 as methyl oleate (compound **11**). In addition, the spectrum displayed 18 carbon fatty chain consisting of signals at δ 1.27 m (28H), 1.63 m (2H) and 2.32 t (2H), a terminal methyl at δ 0.88 t and another methyl ester moiety at δ 3.68. All these signals allowed identification of compound **12** as methyl stearate. The ^{1}H NMR spectrum of ZRP-3 further showed signals similar to compound **12** except the methyl ester group at δ 3.68, indicating the free fatty acid. Thus, the compound **13** was identified as stearic acid [23].

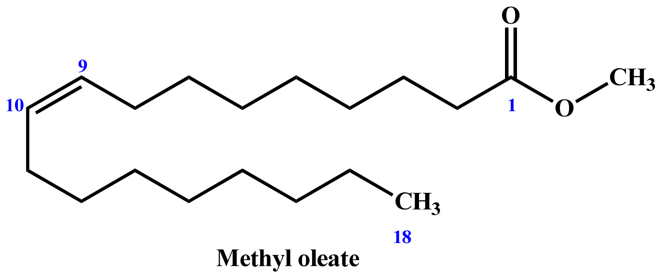

Methyl oleate

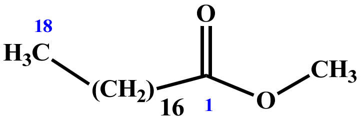

Methyl stearate

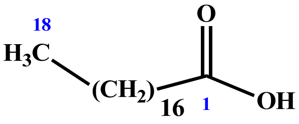

Stearic acid

Table S9. ^{1}H NMR spectral data (500 MHz, $CDCl_3$) of compound 11, 12 and 13 (ZRP-3)

Position	Compound 11<br>δ_H	Compound 12<br>δ_H	Compound 13<br>δ_H
2	2.32 t (2H, *J* = 7.4 Hz)	2.32 t (2H, *J* = 7.4Hz)	2.32 t (2H, *J* = 7.4 Hz)
3	1.63 2H, m	1.63 2H, m	1.63 2H, m
4-17	---	1.27 28H, m	1.27 28H, m
4-7, 12-17	1.27 20H, m	---	---
8, 11	2.03 4H, m	---	---
9, 10	5.36 2H, m	---	---
18-Me	0.88 t (3H, *J* = 6.8 Hz)	0.88 t (3H, *J* = 6.8 Hz)	0.88 t (3H, *J* = 6.8 Hz)
-OMe	3.68 3H, s	3.68 3H, s	---

NMR spectrum of compound 11, 12 and 13 as a mixture of as a mixture of methyl oleate, methyl stearate and stearic acid:

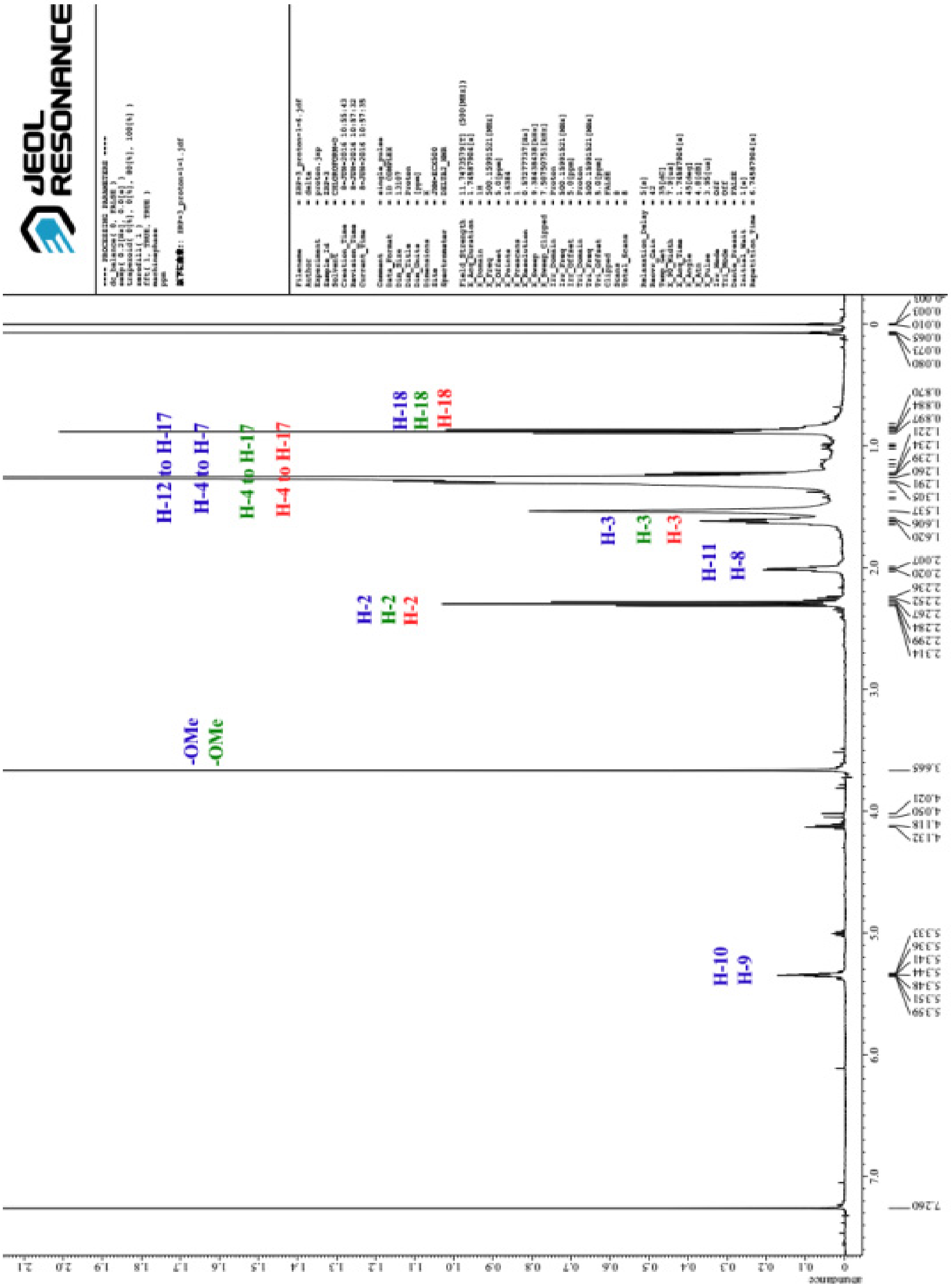

Figure S23. ^{1}H NMR (400 MHz, $CDCl_3$) spectrum values of compound **11**, **12** and **13** (ZRP-3)
